# Visual threat avoidance while host seeking by *Aedes aegypti* mosquitoes

**DOI:** 10.1016/j.celrep.2025.115435

**Published:** 2025-03-19

**Authors:** Geoff T. Meyerhof, Pratik Dhavan, Summer Blunk, Allison Bourd, Ramandeep Singh, Avinash Chandel, Craig Montell

**Affiliations:** 1 Neuroscience Research Institute and Department of Molecular, Cellular Developmental Biology, University of California, Santa Barbara, Santa Barbara, CA 93106, USA

## Abstract

The mosquito *Aedes aegypti* infects hundreds of millions of people annually with disease-causing viruses. When a mosquito approaches a host, the host often swats defensively. Here, we reveal the mosquito’s escape behavior during host seeking in response to a threatening visual cue—a newly appearing shadow. We found that reactions to a shadow are far more aversive when it appears quickly versus slowly. Remarkably, mosquitoes evade shadows under very dim light conditions. Knockout of the TRP channel compromises the ability of mosquitoes to avoid threatening shadows, but only under high light conditions. Conversely, removing two of the five rhodopsins normally present in the compound eyes, Op1 and Op2, diminishes shadow aversion, but only under low light. Upon removal of a threatening visual cue, mosquitoes quickly re-initiate host seeking. Thus, female *Aedes* balance their need to host seek with visual threat avoidance by rapidly transitioning between these two behavioral states.

## In brief

*Aedes aegypti* must weigh their need for blood meals against the danger of being swatted. Meyerhof et al. discover that host-seeking mosquitoes evade a threatening shadow that simulates a swatting individual, even under dim light. They also identify mutations affecting *Aedes* ability to detect shadows under different light conditions.

## Graphical Abstract

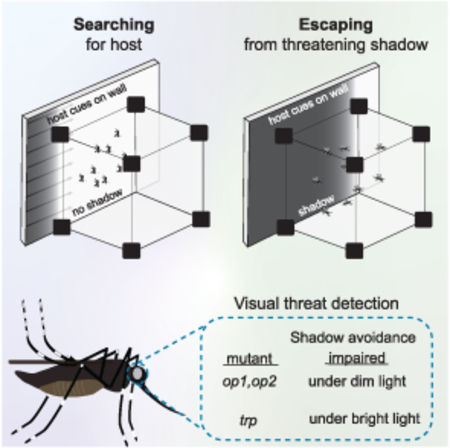

## INTRODUCTION

Female *Aedes* (*Ae.*) *aegypti* mosquitoes spread the pathogens that cause dengue, yellow fever, and other diseases affecting hundreds of millions of people each year. Pathogen transmission occurs because female mosquitoes require a blood meal to produce viable eggs after mating and often bite multiple hosts during their lifetime. In order to find a human host from long- and mid-range distances, mosquitoes use a combination of sensory cues including carbon dioxide, body odors, visual cues, and body heat in the form of infrared radiation.^1–3^ At a close range (<10 cm), female mosquitoes locate their target with additional cues, such as conductive/convective heat, humidity, and taste. Female mosquitoes also derive nutrients from nectar feeding, while male mosquitoes exclusively feed on nectar.^4^

In addition to finding food, mosquito survival also depends on evading threats from predators such as dragonflies^5^ and bats.^6^ The nocturnal mosquito *Anopheles coluzzii* detects moving threats in darkness by sensing the air movements they produce, rather than by employing vision.^7,8^ In contrast, *Ae. aegypti*, which is more active during the daytime, relies largely on vision to escape inbound threats.^8,9^ Both species use erratic flight to slip predators.^8^

The survival of female mosquitoes is further complicated. In their quest for a blood meal, females must weigh their attraction to human cues against the danger of aggression from an unwilling host. In this endeavor, the furtive female mosquito must decide whether the risks from an attack by the very host it aims to bite, outweighs the nutritional and reproductive benefits from blood. Therefore, female mosquitoes must monitor their environment for particularly threatening cues, like rapidly appearing shadows produced by a fast-moving hand, a swishing tail, or an object wielded by a discontented host.^10^ Currently, the genes that underlie the mosquito’s ability to detect visual threats in their environment are unknown. Moreover, it is unclear how the optical properties of a visual threat contribute to a mosquito’s propensity to initiate an escape maneuver.

To see their environment, *Ae. aegypti* are equipped with two compound eyes, each of which is composed of an array of ~350 ommatidia. An individual ommatidium contains eight photoreceptor cells (R1–8),^11^ which are light activated upon photon capture by rhodopsins. In the distantly related dipteran, *Drosophila melanogaster*, the phototransduction cascade culminates with the opening of two cation channels, TRP and TRPL.^12^ Whether *Aedes* TRP channels function in a similar fashion in phototransduction is unknown.

In this work, we investigated the interplay between host-seeking behavior and evasive flight responses in *Ae. aegypti*. We demonstrate that female mosquitoes momentarily forgo their host-seeking behavior when confronted with a visual threat, simulated by a rapidly moving shadow. The velocity of an inbound shadow, and whether the shadow appears or is removed, predicts its aversiveness. Fast, light-to-dark shadows were most likely to stimulate an escape response. We also show that *Ae. aegypti* require Opsin 1 (Op1) and Opsin 2 (Op2) to detect visual threats, but only under low light intensities. We generated mutations disrupting the *Aedes* TRP channel, which impairs threat detection under high light intensities, but leaves low-light threat detection largely intact. Together, these data reveal a conserved role for TRP in *Ae. aegypti* vision and provide insights into how mosquitoes weigh their drive to host seek against their desire to elude a deadly attack by the same host.

## RESULTS

### Design of apparatus to assay responses to an approaching shadow

**Figure 1.**
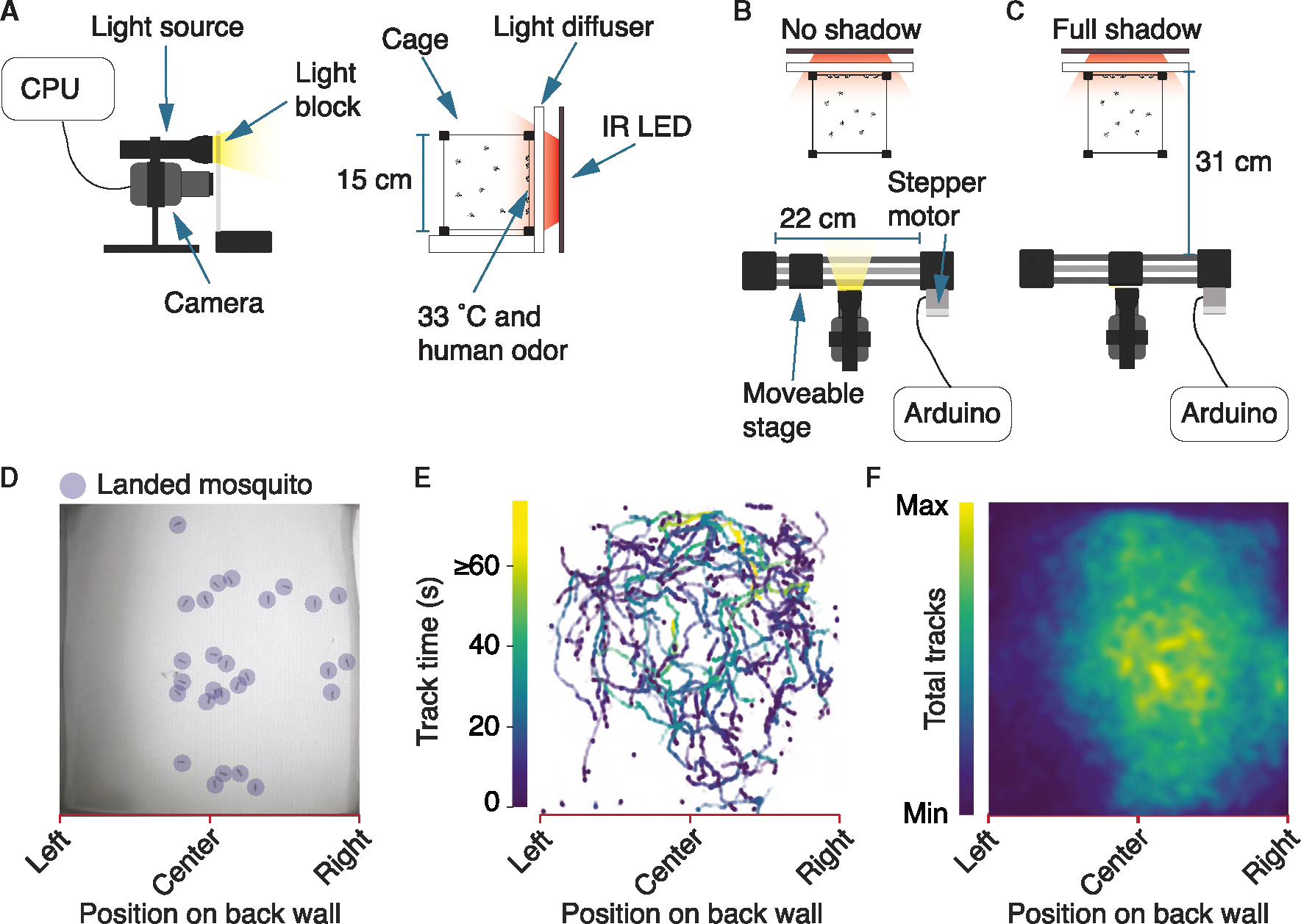
Experimental setup for testing response to visual threats (A) Profile of the setup. The cage is filmed with a webcam with an IR pass filter. Each cage contains ≥30 mosquitoes, which are attracted to the back wall owing to human odor and 37°C heat emanating from IR lights, which dissipates to 33°C at the cage surface. (B) Top view of setup without the shadow. The Arduino microcontroller receives commands from the CPU, then sends commands to the stepper motor to laterally move a light blocker along its track, which when occluding the light source, casts a shadow. (C) Top view of setup with a full shadow, when the light blocker is directly in front of the light source. (D) Sample video frame displaying mosquitoes landed on the back wall of the cage. Landing events, behavior, and takeoffs were scored using custom video tracking. (E) Mosquito track times (single recording). Most tracks are <25 s. (F) Heatmap of tracks (72 mosquito trials, each 150 s). See also Figure S1.

To study the trade-off between host seeking and evasion of a threatening visual cue, we designed a custom behavioral arena that presented mosquitoes with attractive, host-associated cues as well as aversive visual stimuli, and quantified their behavioral responses via video-tracking (Figures 1A–1C, S1A and S1B). We introduced 30 female *Ae. aegypti* into 15 cm^3^ mesh cages, which we modified by replacing the front mesh with a clear plexiglass sheet through which we could film the mosquitoes illuminated with near infrared (IR; 850 nm; Figure 1A). Light of 850 nm is not detected by mosquitoes since it is beyond the maximum wavelength that can activate any rhodopsin.

The back wall of the cage attracted mosquitoes owing to human odor from a worn glove as well as 37°C heat emanating from IR LEDs, which dissipated to 33°C at the inside surface of the cage (ambient temperature: 27°–29.5°C). The 33°C approximates the surface temperature of human skin and provides all three forms of heat transfer (conduction, convection, and radiant). The combination of human odor and three forms of heat transfer resulted in mosquitoes landing on the back wall of the cage (Figure 1D), often followed by bouts of walking (Figures 1D–1F) as they searched for a blood meal.

To simulate a visual threat, we cast a transient rectangular shadow over the entire arena. We controlled the appearance of the shadow by programming a motorized stage to laterally move a light blocker back and forth, which when obstructing the light cast a shadow over the entire arena (Figure 1B, no shadow; Figure 1C, full shadow). The light blocker consisted of a clear plexiglass sheet affixed to the moveable stage, with a rectangular strip of opaque film secured to the top of the plexiglass, which blocked visible light when the film passed in front of the light source. We videotaped each experiment and developed custom video-tracking scripts to unbiasedly annotate the mosquitoes’ behavior along the back wall of the cage, as well as their response to the visual threat created by the shadow (Figures S1A, S1B and Video S1).

### A fast-moving shadow temporarily suspends host seeking and initiates takeoffs

**Figure 2.**
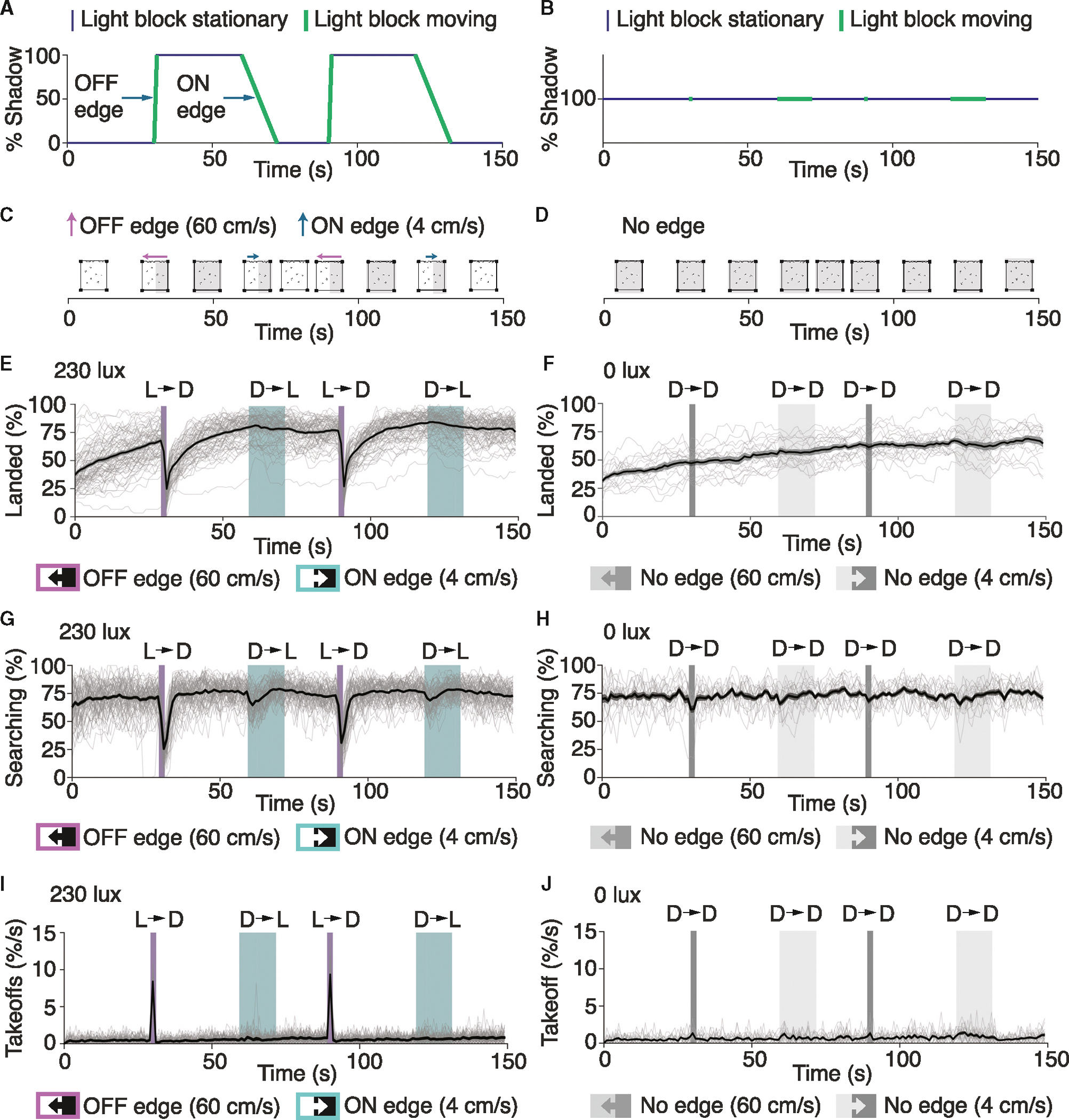
Behavioral responses to a visual threat in mosquito cages (A and B) Shadow-movement pattern on the back wall. (A) 230 lux. (B) 0 lux. (C) Vector of shadow movement (230 lux). Rectangular OFF-edge shadow (60 cm/s) at times 30 and 90 s, and an ON-edge shadow (4 cm/s) at 60 and 120 s (D) Vector of shadow movement (0 lux). No shadow cast at 0 lux. (E) Percentage of mosquitoes landed on the back wall (230 lux). Purple shading, 60 cm/s OFF-edge shadow. Green shading, 4 cm/s ON-edge shadow. (F) Percentage of mosquitoes landed on the back wall (0 lux). Narrow gray shading, 60 cm/s movement of blocker. Wide gray shading, 4 cm/s movement of blocker. (G and H) Percentage of landed mosquitoes searching along the back wall. (G) 230 lux. (H) 0 lux. (I and J) Percentage of total takeoffs/second. (I) 230 lux. (J) 0 lux. (E, G, and I) Black traces, averages from 72 trials; gray traces, results from each individual trial. (F, H, and J) Black lines, averages from 18 trials; gray lines, results from each individual trial. Dark gray shading denotes fast light blocker movements. Light gray shading denotes slow light blocker movements. See also Figure S2

To characterize the response of a simulated visual threat while host seeking, we recorded 72 trials from 24 groups of 30 female mosquitoes. We conducted these experiments under 230 lux white light (0.20 mW/cm^2^, Figure S1C), similar to the light intensity at early dawn or late evening when *Ae. aegypti* are most active.^13^ To assess the impact of shadow speed on behavior, we subjected the mosquitoes to two fast-moving (60 cm/s) light-to-dark shadows and two slow-moving (4 cm/s) dark-to-light shadows during each 150-s recording (Figures 2A and S1D). Both shadow movements produced a single moving edge, with opposite polarities for the mosquitoes depending on the direction of the shadow. The light-to-dark shadow created an OFF-edge as the illuminance decreased and the dark-to-light shadow created an ON-edge as the illuminance increased. As a control, we housed different mosquitoes under identical experimental conditions (identical light blocker movement pattern) at 0 lux (Figure 2B). After 30 s of acclimation to the arena, the mosquitoes housed at 230 lux experienced a fast (60 cm/s) OFF-edge shadow that covered the entire back wall of the cage (Figure 2C). The mosquitoes remained in the dark for an additional 30 s, after which we slowly reset the light blocker, casting a slow 4 cm/s ON-edge shadow. The mosquitoes housed at 0 lux experienced no shadow as the stage moved back and forth (Figures 2B and 2D).

Over 50% of the mosquitoes landed on the back wall of the cage in <1 min, either in the dark (0 lux) or when exposed to 230 lux light (Figures 2E and 2F). Once landed, they often engaged in short bouts of walking and probing through the mesh (Figures 1D and Video S2). This “searching” behavior began almost instantaneously when the mosquitoes were placed in the cage, and at any time ~70% of mosquitoes on the back wall were host seeking under either condition (Figures 2G and 2H). Mosquitoes in the light or dark spent nearly identical amounts of time on the back wall (Figure S2A) as if searching for blood meals (Figure S2B). These data demonstrate that darkness does not diminish the attractive combination of human odor and all three forms of heat.

Upon movement of the light blocker, we observed substantial differences in behavior depending on whether the females were exposed to light or were in the dark. At 230 lux, the fast OFF-edge shadow (60 cm/s) initiated at 30 and 90 s rapidly reduced the number of mosquitoes that landed on the back wall (Figures 2E, 2I, and Video S3). After the visual threat was removed, >60% of the mosquitoes re-landed within 20 s (Figure 2E). Among the landed mosquitoes, ~70% were searching for blood meals (Figure 2G). These data demonstrate that visual threats only transiently interrupt the drive to host seek. In contrast, mosquitoes housed in the dark did not take off in response to the fast-stage movement (Figures 2F, 2J, and Video S4), confirming that the decision to abandon host seeking and takeoff required visual input. Interestingly, mosquitoes that did not take off in response to the fast light-to-dark shadows presented at 30 and 90 s temporarily paused their searching behavior (Figure 2G). This cessation of searching did not occur among mosquitoes maintained in the dark (Figure 2H, 30 and 90 s), again indicating that visual cues, rather than auditory or vibration stimuli created by the movement of the light blocker, reduced the proclivity to actively search for blood meals.

Taken together, our results demonstrate that a visual threat simulated by a fast-moving shadow causes the mosquitoes to either become stationary or take off. This freeze or flight response to visual threats is reminiscent of what others have observed in distantly related dipterans, such as *Drosophila melanogaster*.^14^

### Stationary and searching *Aedes* are equally prone to takeoff in response to a visual threat

**Figure 3.**
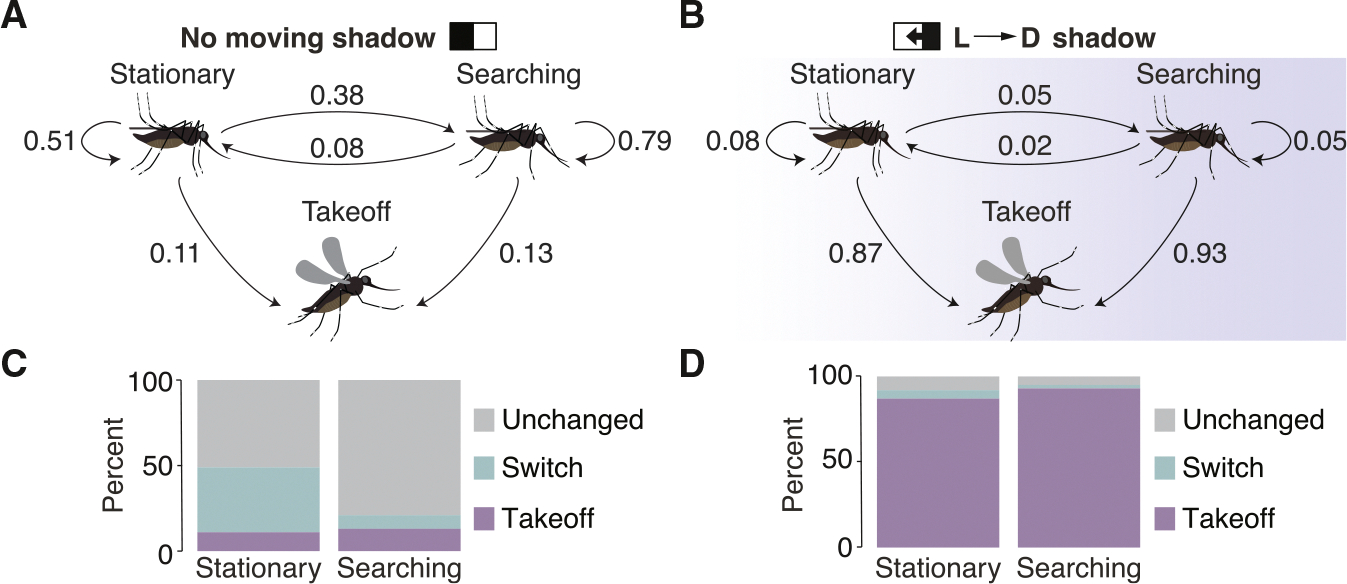
Behavioral transition probabilities without a shadow, and during a fast light-to-dark shadow (A) Behavioral transition probabilities in the absence of a light-to-dark shadow (calculated over a 2-s window). (B) Behavioral transition probabilities during a light-to-dark shadow. (C) Percentage of times the mosquitoes made the transitions shown in (A). (D) Percentage of times the mosquitoes made the transitions shown in (B).

Next, we characterized the relative takeoff behavior of stationary and host-seeking mosquitoes by examining the responses to a rapid OFF-edge or transitioning between being stationary and initiating a bout of searching, and vice versa. Because each stationary bout lasted for 2.1 ± 0.3 s (Figure S2C), we used 2 s to calculate the probability of a mosquito transitioning between behaviors (see STAR Methods). As a control, we first examined mosquitoes experiencing a static visual scene (i.e., no shadow), and found that if they were stationary or searching, they most commonly sustained those behaviors (Figures 3A and 3C; 51% chance of remaining stationary and 79% chance of remaining searching). On average, it was more likely for a stationary mosquito to begin searching (38% chance) than for a searching mosquito to become stationary (8% chance). Both stationary and searching mosquitoes had a similar spontaneous takeoff rate (11% and 13% chance, respectively; Figures 3A and 3C).

The behavioral transition probabilities during a fast OFF-edge shadow were dramatically different from those exposed to a static visual scene (Figures 3A vs. 3B). During a 2-s interval of a fast-moving shadow, mosquitoes rarely maintained stationary or searching, and they seldom transitioned between these states (Figures 3C vs. 3D; 1% stationary to searching; 2% searching to stationary). Instead, takeoffs predominated (87% and 93% chance of a stationary or searching mosquito initiating an escape, respectively; Figure 3D). This indicates that regardless of whether a female is actively searching for a blood meal or stationary on a surface with host cues, there is a nearly equal likelihood that they will respond to a visual threat by initiating a takeoff.

### Shadows rather than rapid changes to light intensity are most startling to *Aedes*

**Figure 4.**
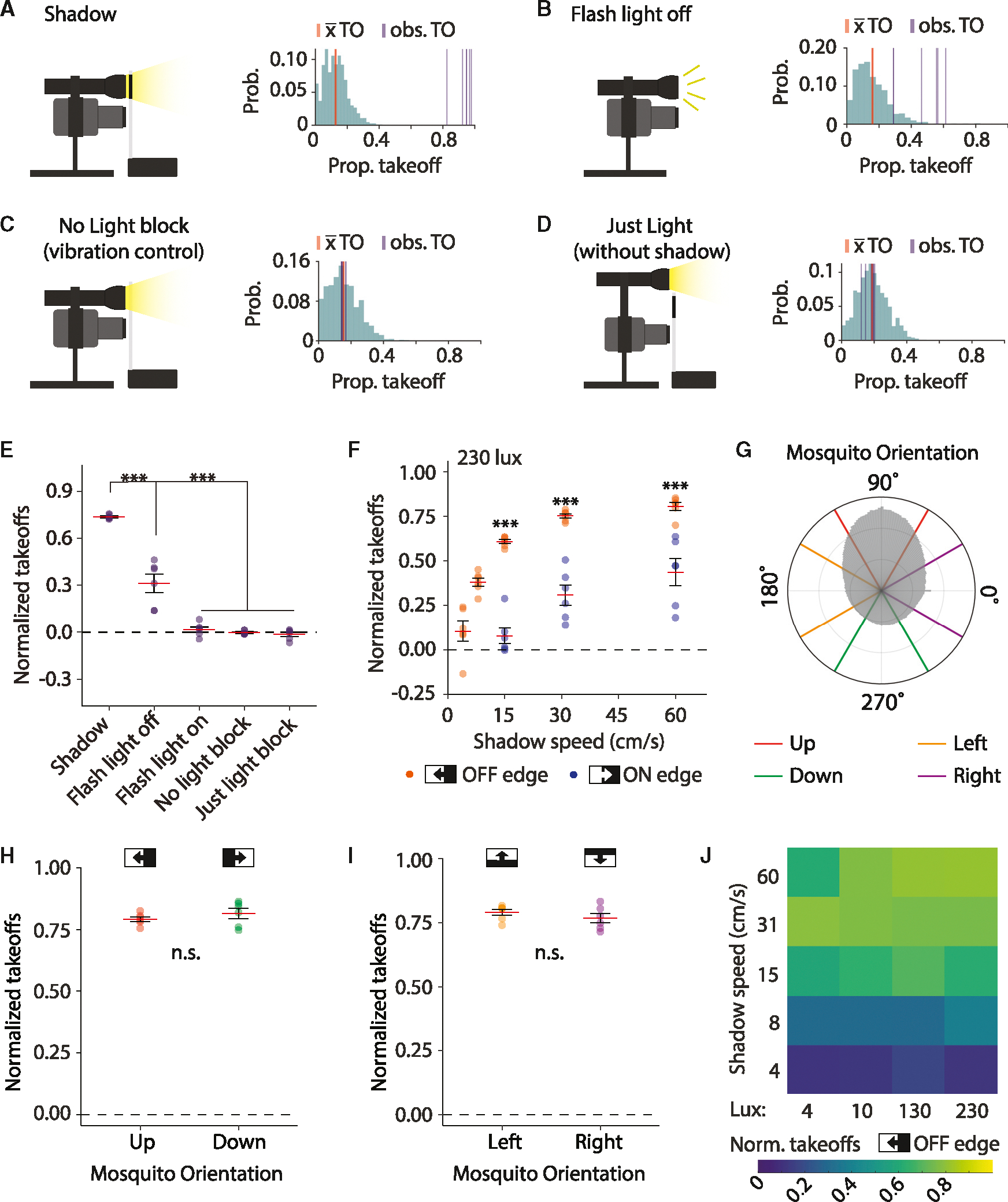
Fast light-to-dark shadows trigger an escape response (A–D) (Left) Cartoons of setups for the control experiments. (Right) Distributions of spontaneous takeoffs from 20,000 random samples in each videos set (see STAR Methods). The mean spontaneous takeoff rate ($\bar{\text{x}}$ TO; red) was determined via random sampling. The observed takeoff rate (obs. TO; purple) is the takeoff rate in response to the stimulus. Normalized takeoffs represent the difference between $\bar{\text{x}}$ TO and obs. TO. (A) Shadow-movement experiment. (B) Flash light off. (C) No light block (LB). (D) LB apparatus moved but positioned so that no shadow is cast. (E) Normalized takeoffs from protocols depicted in (A–D). One-way ANOVA (*p<* 0.001) followed by Tukey’s honestly significant difference (HSD) test. (F) Effects of shadow speed and polarity on takeoffs. ON-edge shadows (blue dots), and OFF-edge shadows (orange dots). Speeds tested for ON- and OFF-edge shadows: 60, 31, and 15 cm/s. Additional speeds for OFF-edge shadows: 8 and 4 cm/s. One-way ANOVA (*p* < 0.001) followed by Tukey’s HSD test. (E–F) *n* = six cages with 30 mosquitoes/condition, each recorded three times. (G) Polar histogram displaying probability of mosquito orientation on the back cage wall. Orientations subdivided into four groups each spanning 60° to specify mosquitoes facing up, down, left, or right. Probabilities from 29,312 landing events from 36 cages of 30 mosquitoes. (H) Normalized takeoffs by mosquitoes facing up vs. down. (I) Normalized takeoffs by mosquitoes facing left versus right. (H and I), Two-tailed unpaired Student’s *t* tests. *p* > 0.05. (G–I), *n* = 6, each consisting of six cages of 30 mosquitoes measured three times each. (J) Heatmap displaying effects of shadow speed and light intensity on normalized takeoffs (Norm. takeoffs) for OFF-edge shadows. Columns, light intensity. Rows, shadow speed. *n* = 6 cages of 30 mosquitoes per condition, each recorded three times. ***p < 0.001. Means ± SEMs. See also Figure S2.

Although we observed that rapid OFF-edge shadows readily triggered takeoff responses in female *Aedes* (Figure 2I), other cues associated with the moving light blocker might contribute to this behavior. Therefore, we tested the impact of a rapid change in light intensity without a shadow, the sound vibrations associated with the moving stage without a shadow, and the sight of the moving light blocker itself rather than the shadow that it produces. To do so, we calculated a “normalized takeoffs” metric by recording the number of mosquitoes that were present on the back wall 500 ms before we presented a stimulus and then calculated the proportion that took off either during the stimulus or ≤1 s after the stimulus (denoted as observed [obs.] takeoffs [TO]; Figures 4A–4D). We then normalized the observed takeoffs to the spontaneous takeoff rate (denoted as $\bar{\text{x}}$ TO; Figures 4A–4D) in a given trial (see STAR Methods). We found that the spontaneous takeoff rates were relatively consistent over the span of an experiment, with ~13% taking off from the back wall every ~2 s (Figure S2D). The spontaneous takeoff rate ($\bar{\text{x}}$ TO) was similar in each of the conditions tested: positive control, which produces a shadow (Figure 4A, 0.13), light flashed from on to off, but no shadow produced (Figure 4B, 0.15), moving apparatus with no light block to test the effect of vibration alone (Figure 4C, 0.15), and moving apparatus, but no shadow produced (Figure 4D, 0.18). A “normalized takeoffs” value of 0 occurs if the response to a given stimulus was identical to their spontaneous takeoff rate, whereas a value above 0 indicates an increased takeoff rate, and a value below 0 indicates the opposite.

To test whether a rapid change in light intensity without a moving shadow triggers takeoffs, we maintained mosquitoes under 230 lux, and programmed the lights to flash from on to off at 30 and 90 s (Figure 4B, flash light off), and from off to on at 60 and 120 s. As a positive control, we subjected the mosquitoes to the fast-moving (60 cm/s) OFF-edge shadow, which induced 98.0% ± 0.6% takeoffs (Figures 4A and 4E; normalized takeoffs = 0.74 ± 0.01). Flashing the lights off (lights turning off without a shadow) also induced takeoffs (Figure 4B). However, the percentage of responding mosquitoes (46.3% ± 5.8%; normalized takeoffs = 0.31 ± 0.06) was significantly lower than the positive control (Figure 4E). Remarkably, flashing the lights on elicited almost no response (Figure 4E; normalized takeoffs = 0.02 ± 0.02).

To determine whether the mosquitoes were responding to the sound or vibrations from the moving stage rather than the shadow, we removed the light blocker so that the stage moved without casting a shadow (Figure 4C, no light block). We also tested whether mosquitoes were responding to the sight of the moving light blocker (an opaque white rectangle), rather than the shadow it cast, by positioning the light source above the light blocker, so that when the stage moved, no shadow was cast but the moving light blocker remained visible to the mosquitoes (Figure 4D, light block but no shadow). These conditions dramatically reduced the number of takeoffs to 15.1% ± 0.6% (no light block) and 17.4% ± 1.3% (just light block), which was practically identical to their spontaneous takeoff rate (normalized takeoffs: 0.00 ± 0.01 and 0.01 ± 0.01, respectively; Figure 4E). Shadow-induced takeoffs were unchanged among technical replicates run on the same cage (Figure S2E). These data reveal that the mosquitoes were not responding to vibration, sound, or secondary visual cues produced by the movement of the light blocker. Thus, visual cues involving a moving shadow are most likely to induce an escape response.

### Speed of moving shadow impacts the escape response

The onset of an OFF-edge shadow might be more aversive than an ON-edge shadow as the appearance of a shadow is more likely to reflect an approaching predator. To further address the relative effects of an OFF-edge vs. an ON-edge shadow, we employed two paradigms. In one set of experiments, the mosquitoes began in the light, experiencing a fast OFF-edge at times 30 and 90 s, with a slow ON-edge shadow at 60 and 120 s (Figure S2F). In a second set of experiments, the mosquitoes began in the dark, and experienced a fast ON-edge shadow and a slow OFF-edge shadow at opposite times (Figure S2G). We observed many more takeoffs in response to a fast OFF-edge shadow (light to dark, 60 cm/s) relative to rapid ON-edge shadow (dark to light, 60 cm/s; Figure 4F). We then subjected mosquitoes to slower shadows: 31 and 15 cm/s. At these speeds, there were only modest declines in response to the OFF-edge shadow from a normalized takeoff rate of 0.81 ± 0.01 at 60 cm/s, to 0.75 ± 0.01 at 31 cm/s, and 0.61 ± 0.01 at 15 cm/s (Figure 4F). However, the ON-edge shadow rapidly became much less aversive when the light transitions occurred at slower speeds. At 15 cm/s, the ON-edge shadow elicited a minimal aversive reaction (Figure 4F; normalized takeoffs = 0.10 ± 0.04). Even at a slower speed of 8 cm/s, the takeoffs from an OFF-edge shadow were substantially more aversive (normalized takeoffs = 0.38 ± 0.02) than the ON-edge shadow traveling at 15 cm/s, and was similarly aversive as the ON-edge shadow moving at 60 cm/s (Figure 4F). Thus, OFF-edge, light-to-dark shadows are much more aversive than ON-edge, dark-to-light shadows.

The speed of an OFF-edge shadow markedly affects takeoff probability, but only when it is very slow. Although slow-moving OFF-edge shadows (Figure S2G) did not readily induce takeoffs, some mosquitoes momentarily froze, remaining stationary for 1.10 ± 0.30 s. Even at the slowest shadow velocity tested (4 cm/s), the most common reaction was to take flight (70% takeoff vs. 30% freeze; Figure S2H).

To determine whether the direction of the shadow relative to the mosquitoes’ orientation affects its aversiveness, we determined the orientation of each mosquito on the back wall of the cage prior to exposing them to an OFF-edge shadow. Overall, the heads of landed mosquitoes were most likely to orient toward the top of the cage (90°; Figure 4G). As the mosquitoes walked and probed through the mesh, a minority faced left (180°), right (0°), or down (270°). We averaged data from six independent experiments and categorized their orientations into four groups: up, down, left, or right. Mosquitoes in each of these groups experienced a shadow moving in a different direction, which together spanned the four cardinal directions of motion. Mosquitoes facing up experienced a left-moving edge and those facing down a right-moving edge. The aversiveness of a shadow did not depend on whether it was moving to the left or to the right relative to the mosquitoes, as insects in both up and down orientations had nearly identical normalized takeoff rates (Figure 4H). Similarly, upward- and downward-moving OFF-edge shadows (detected by mosquitoes facing leftward or rightward, respectively) had no impact on the takeoff response (Figure 4I). Therefore, the direction of an OFF-edge shadow does not impact its aversiveness, as mosquitoes in all orientations were equally likely to initiate escape responses.

### A visual threat causes only a temporary aversion to area with host cues

Host-seeking female mosquitoes must balance the potential danger of a visual threat, such as the rapid onset of a shadow, with the need to seek a host so that they can consume blood. Thus, the question arises whether mosquitoes experiencing a visual threat in a region laced with attractive human cues (human odor and all three forms of heat transfer from a 33°C zone) would continue to avoid the region, or quickly return to the area after the threat disappears. To address this question, we compared the responsiveness to a 60 cm/s OFF-edge shadow that moved from right to left along their cage, stopping when it covered either half or all of the back wall where the mosquitoes were landed. To assess the potential lasting aversiveness of these visual threats, we plotted the average position of the landed mosquitoes (Figure S2I), reasoning that a landing bias toward one-half of the cage after a visual threat would reflect a sustained aversion. Prior to the onset of the OFF-edge, mosquitoes in both conditions were, on average, located in approximately the center of the cage. Immediately, following the right-to-left moving shadow (Figure S2F, purple line at 30 and 90 s), mosquitoes in both groups displayed a slight bias toward the left side of the cage (Figure S2I), which may reflect their having taken off in that direction following the shadow. This slight preference quickly dissipated. In both groups, the mosquitoes were almost equally prone to occupy the right or left side of the cage following a moving shadow (Figure S2I), indicating that a localized visual threat does not create a sustained aversion to a single side of the cage. A shadow covering only half of the cage was significantly less aversive than one spanning the entire back wall, demonstrating that mosquitoes are most prone to takeoff in circumstances where they are entirely covered by the shadow, or where there is a large change in total illuminance (Figure S2J).

### Mosquitoes avoid shadows even in very low-light environments

To determine whether mosquitoes avoid a shadow under low-light conditions, we subjected them to a 60 cm/s OFF-edge shadow at intensities as low as 4 lux and increasing up to 230 lux (0.05 mW/cm^2^ to 0.20 mW/cm^2^; Figure 4J, top row). Remarkably, mosquitoes displayed a robust takeoff response to a moving shadow even at 4 lux (Figure 4J, top row; normalized takeoffs: 0.60 ± 0.02 at 4 lux, 0.81 ± 0.02 at 230 lux). To determine whether there was an interaction between shadow speed and light intensity, we repeated these light intensity experiments at additional shadow speeds: 31, 15, 8, and 4 cm/s (Figure 4J, rows 2–5). Overall, there was a substantial effect from the speed of a shadow (η^2^ = 0.51, *p* < 0.001) and a much smaller effect from the ambient light intensity (η^2^ = 0.02, *p* = 0.04), with an insignificant interaction between the two factors (η^2^ = 0.01, *p* > 0.05). We conclude that the speed of an OFF-edge shadow significantly impacts aversiveness at all lux levels tested. Our data also highlight the sensitivity of the *Aedes* visual system, which can detect threats even under very low-light conditions.

### Males and non-host-seeking females also respond to visual threats by initiating takeoffs

**Figure 5.**
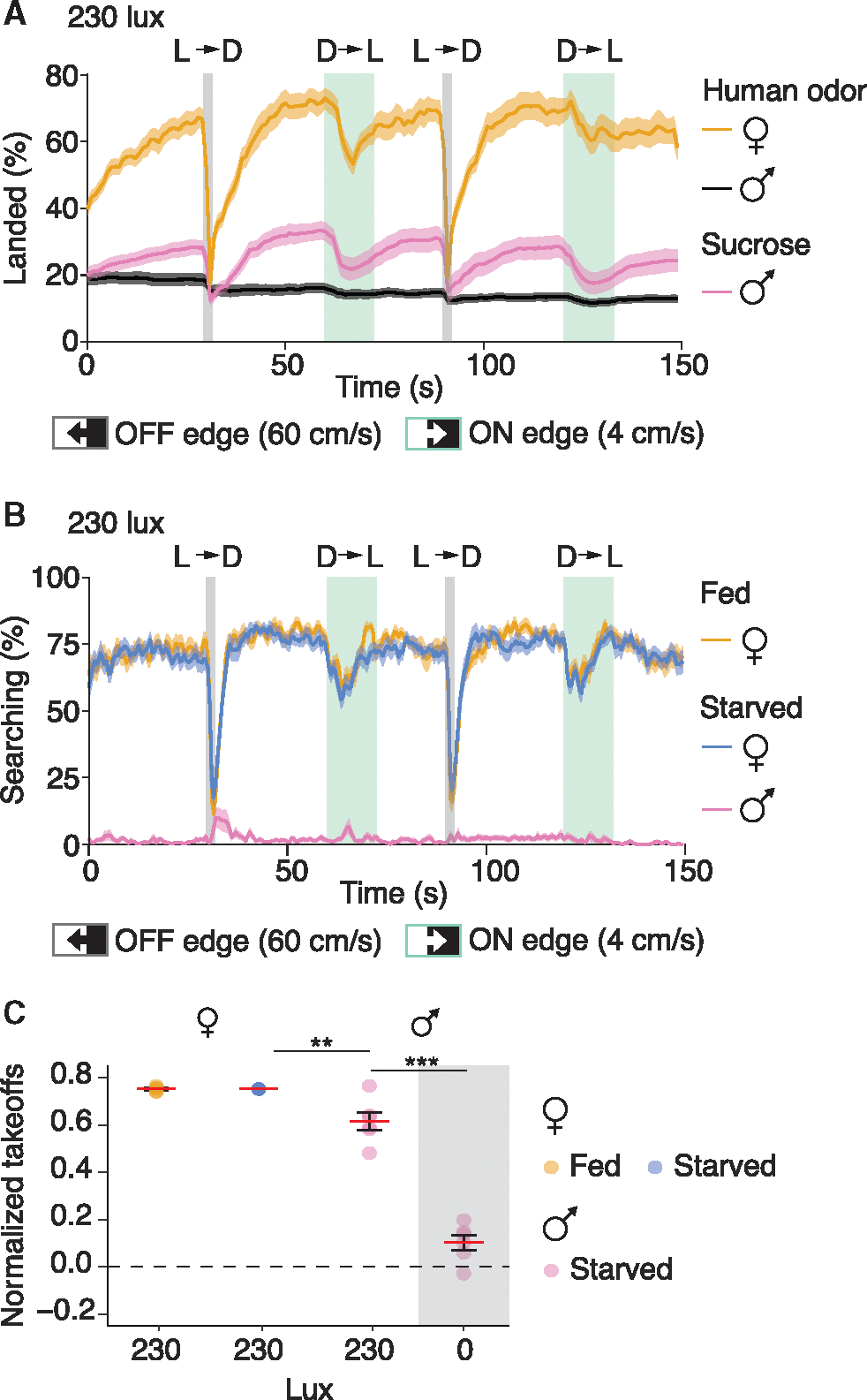
Males respond to visual threats by initiating takeoffs (A) Percentages of males and females that landed on the back wall of cages laced either with human odor or 10% sucrose. The back walls were all exposed to 33°C heat from IR lights. Orange trace, females presented with human odor. Black trace, males presented with human odor. Pink trace, males presented with 10% sucrose. (B) Percentage of landed mosquitoes searching along the back wall. Orange trace, fed females. Blue trace, 16-h starved females. Pink trace, males attracted to the back wall with 10% sucrose. (C) Normalized takeoffs from fast-moving light-to-dark (OFF-edge) shadows (60 cm/s) for males (starved) in the dark (0 lux) or exposed to 230 lux, or females (fed or starved) exposed to 230 lux. One-way ANOVA (*p* < 0.001) with Tukey’s honestly significant difference post hoc test. *n* = 6 cages each measured three times. **p < 0.01. ***p < 0.001. Means ± SEMs. See also Figure S3.

There are reports that male *Ae. aegypti* are attracted to human odors, perhaps as a means to locate females.^15^ With this in mind, we measured the male response to a moving shadow in the presence of host cues emanating from the back wall of the cage. Unlike females, which readily responded to the human odor and heat in our assay, the male participation rate was much lower (<19% males, black trace; >70% females, yellow trace; Figure 5A). Between 0 and 30 s, the percentage of females participating in the assay increased from ~40% to ~70% and rebounded after the mosquitoes took off in response to a shadow (Figure 5A). However, the participation rate from males decreased slightly as the experiment progressed (Figure 5A), suggesting that the mosquitoes that initially landed on the back wall were there by chance, rather than as a result of their attraction to human odor and the 33°C heat, consistent with past observations.^16^

To entice males to participate in the assay by landing on the back wall, we starved them for 16 h and applied a 10% sucrose solution to the back of the cage. This increased their participation rate to 33% (Figure 5A, pink trace). The number of males that landed on the back wall of the cage rebounded after shadow-induced takeoffs, indicating an attraction to the sucrose solution (Figure 5A, pink trace). Unlike females, which initiated bouts of walking and probing (i.e., searching) after landing on the back wall of the cage, male mosquitoes were overwhelmingly stationary after landing (Figure 5B). This effect was unlikely due to starvation, as upon exposure to host cues, equivalently starved females exhibited an equal propensity to search compared with the unstarved control (Figure 5B, blue vs. orange traces). Rather, walking and probing are sexually dimorphic responses to attractive host cues. However, like females, males that landed readily responded to a 60 cm/s OFF-edge shadow (Figure 5C; normalized takeoffs: 0.62 ± 0.04 at 230 lux, and 0.10 ± 0.03 at 0 lux), albeit at a somewhat reduced level compared with females (Figure 5C; normalized takeoff: 0.75 ± 0.00).

To test whether the slightly reduced OFF-edge response in males relative to females was due to the absence of host seeking, we attracted starved females to the back wall of the cage with sucrose but did not provide any host-seeking cues (see STAR Methods). This resulted in females landing on the back wall of the cage and remaining stationary (Figures S3A and S3B; 1.7% ± 0.4% searching in sucrose vs. 68.7% ± 2.5% searching with host cues). Intriguingly, when confronted with a shadow, the females attracted to sucrose also initiated takeoffs (Figure S3C; Normalized takeoffs = 0.57 ± 0.06), but at a somewhat reduced level compared with females responding to host cues (Figure S3C; Normalized takeoffs = 0.78 ± 0.01). Together, these data demonstrate that the responses to visual threats are relatively consistent between males and females, but that host cues modestly increase females’ sensitivity to a visual threat.

### TRP channel required for normal visual threat detection under higher light levels

Due to the detection of a fictive predator through the sense of vision, we set out to assess the impact of mutations that diminish vision on the sensitivity of shadow detection. In the fruit fly, *Drosophila melanogaster*, the phototransduction cascade culminates with opening of the TRP and TRPL cation channels.^12^ Mutations in *Drosophila trp* severely compromise phototransduction, eliminating the ability to sustain photoreceptor depolarization at high light intensities; while, at low-light intensities the light response is maintained.^17,18^

**Figure 6.**
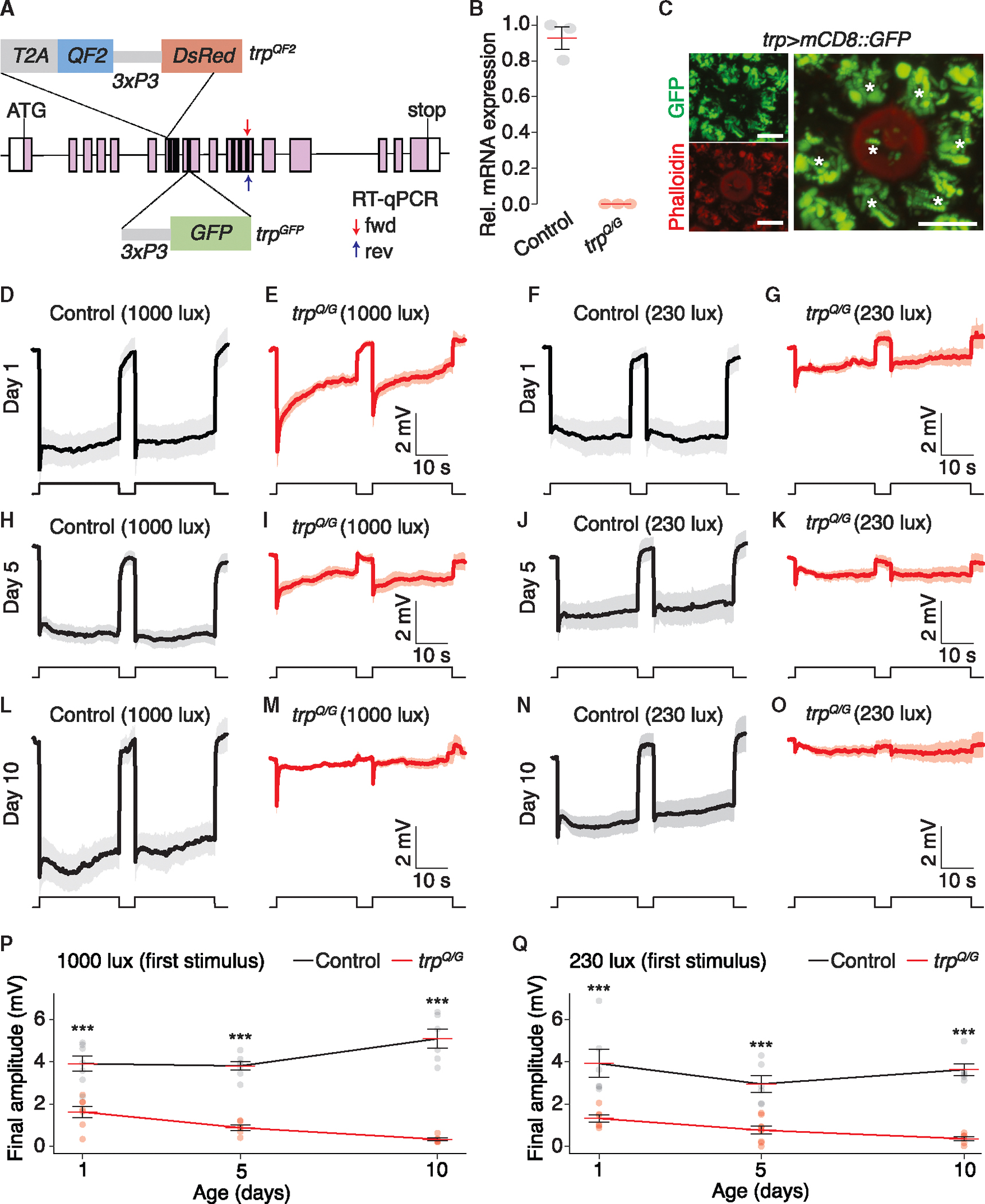
Function of *Ae. aegypti* TRP in the light response (A) Cartoons of *trp*^*QF2*^ and *trp*^*GFP*^. The black vertical lines represent transmembrane domains. The positions of the forward (fwd) and reverse (rev) primers used for the RT-qPCR are indicated. (B) Relative *trp* mRNA expression in control and *trp*^*Q/G*^ measured by RT-qPCR. *n* = 3 biological replicates. (C) *trp* reporter expression in an ommatidium from a *trp*^*QF2*^>*mCD8::GFP* retina viewed by confocal microscopy: GFP (green), phalloidin (red). The asterisks indicate cell bodies of photoreceptor cells visualized with *mCD8::GPF*. The phalloidin labels the fused rhabdomeres. 13-μm-thick optical section. Scale bars, 6 μm. (D–O) ERGs in response to the indicated light intensities. The ages and genotypes of the mosquitoes are indicated. Mosquitoes were exposed to two 25-s pulses of light, separated by 5-s dark adaptation. The shading indicates means ± SEMs. *n=* 6–7. (D) One-day-old control. 1,000 lux. (E) One-day-old *trp*^*Q/G*^. 1,000 lux. (F) One-day-old control. 230 lux. (G) One-day-old *trp*^*Q/G*^. 230 lux. (H) Five-day-old control. 1,000 lux. (I) Five-day-old *trp*^*Q/G*^. 1,000 lux. (J) Five-day-old control. 230 lux. (K) One-day-old *trp*^*Q/G*^. 230 lux. (L) Ten-day-old control. 1,000 lux. (M) Ten-day-old *trp*^*Q/G*^. 1,000 lux. (N) Ten-day-old control. 230 lux. (O) Ten-day-old *trp*^*Q/G*^. 230 lux. (P and Q) Final ERG amplitudes from first light stimulus for the traces shown in (D–O). Two-way ANOVA with factors of age (P: *p* > 0.05; Q: *p<* 0.05), genotype (P: *p* < 0.001; Q: *p* < 0.001), and age:genotype interaction (P: *p <* 0.001; Q: *p* > 0.05). Group means compared via Tukey’s honestly significant difference test. ***p < 0.001. Means ± SEMs. See also Figure S4.

To test the impact of eliminating *Ae. aegypti* TRP (79.2% amino acid identity with *Drosophila* TRP) on the aversive response to a shadow, we generated two alleles using CRISPR-Cas9 (Figure 6A; see STAR Methods), one of which included a *QF2* gene reporter (*trp*^*QF2*^). The mutations prevented detectable expression of the *trp* transcripts by RT-qPCR (Figures 6B and S4A). To examine expression of the *trp* reporter, we used the *trp*^*QF2*^ allele to drive expression of *QUAS-mCD8:: GFP*. As a marker for the rhabdomeres, which contains most of the actin in photoreceptor cells, we co-stained the tissue with phalloidin. Due to the fused rhabdomeres in *Aedes* ommatidia,^11^ the phalloidin labeled a ring near the center of the ommatidia (Figure 6C). The GFP marked the six outer photoreceptor cells (Figure 6C, asterisks). In addition, there was some GFP expression near the center of the ommatidia, which presumably corresponds to R7 and R8 photoreceptors or both (Figure 6C). Although mCD8:GFP should label the photoreceptor cell membranes, and therefore be concentrated in the rhabdomeres, the staining was almost exclusively in the cell bodies of the photoreceptor cells (Figure 6C), possibly due to an impairment of the fusion protein in exiting the endoplasmic reticulum.

We assessed the effect of mutating *trp* on the light response by performing electroretinogram (ERG) recordings, which measure the summed responses of all retinal cells to light. We dark-adapted 1 day-old control and transheterozygous *trp* mutant mosquitoes (*trp*^*QF2*^/*trp*^*GFP*^; referred to as *trp*^*Q/G*^) for 2 min, and then exposed them to two pulses of 1,000 lux light. Control mosquitoes exhibited an initial decline during the first ~60 ms, and then maintained the corneal negative receptor potential for the remaining duration of each light stimulus (Figure 6D). The *trp*^*Q/G*^ light responses diminished to levels that were 42% (first pulse) and 34% (second pulse) relative to the control, prior to termination of the light stimuli (Figures 6D and 6E). In contrast to the ERG response of the *Drosophila trp* mutant, which declines to baseline within a couple of seconds of exposure to bright light,^18–20^ the light response was not eliminated in *trp*^*Q/G*^ mosquitoes (Figure 6E), even after 90 s of light (Figures S5A and S5B). At 230 lux, the ERG responses of wild-type mosquitoes were similar to their responses at 1,000 lux (Figures 6D and 6F). At 230 lux, the *trp*^*Q/G*^ mutant response did not show a decline in amplitude (Figure 6G), consistent with the *Drosophila trp* mutant, which only shows a transient ERG upon exposure to very bright light.^20^ The ERG amplitudes of 5- and 10-day-old controls were similar to 1-day-old mosquitoes at both light intensities (Figures 6D, 6F, 6H, 6J, 6L, 6N, 6P, and 6Q). However, the amplitude of the *trp*^*Q/G*^ ERGs diminished with age (Figures 6E, 6G, 6I, 6K, 6M, 6O, 6P, and 6Q).

To determine whether the age-dependent decline in the *trp* ERG responses was due to retinal degeneration, we imaged the ultrastructure of the compound eyes. Each *Ae. aegypti* compound eye is composed of ~350 ommatidia, consisting of eight photoreceptor cells. Phototransduction takes place in the microvillar portion of the photoreceptor cells—the rhabdomeres. The rhabdomeres are fused, and only seven of the eight rhabdomeres are present in the distal sections of each rhabdomere. In 5-day-old mosquitoes, the morphology of the rhabdomeres was comparable between wild-type and the *trp*^*Q/G*^ mutant (Figures S4B and S4C). Therefore, the decreased ERG amplitudes exhibited by up to 5-day-old *trp*^*Q/G*^ mosquitoes did not appear to be due to retinal degeneration. By 10 days of age, the *trp*^*Q/G*^ mutant underwent severe loss of the rhabdomeres, which is in contrast to wild-type (Figures S4D and S4E).

**Figure 7.**
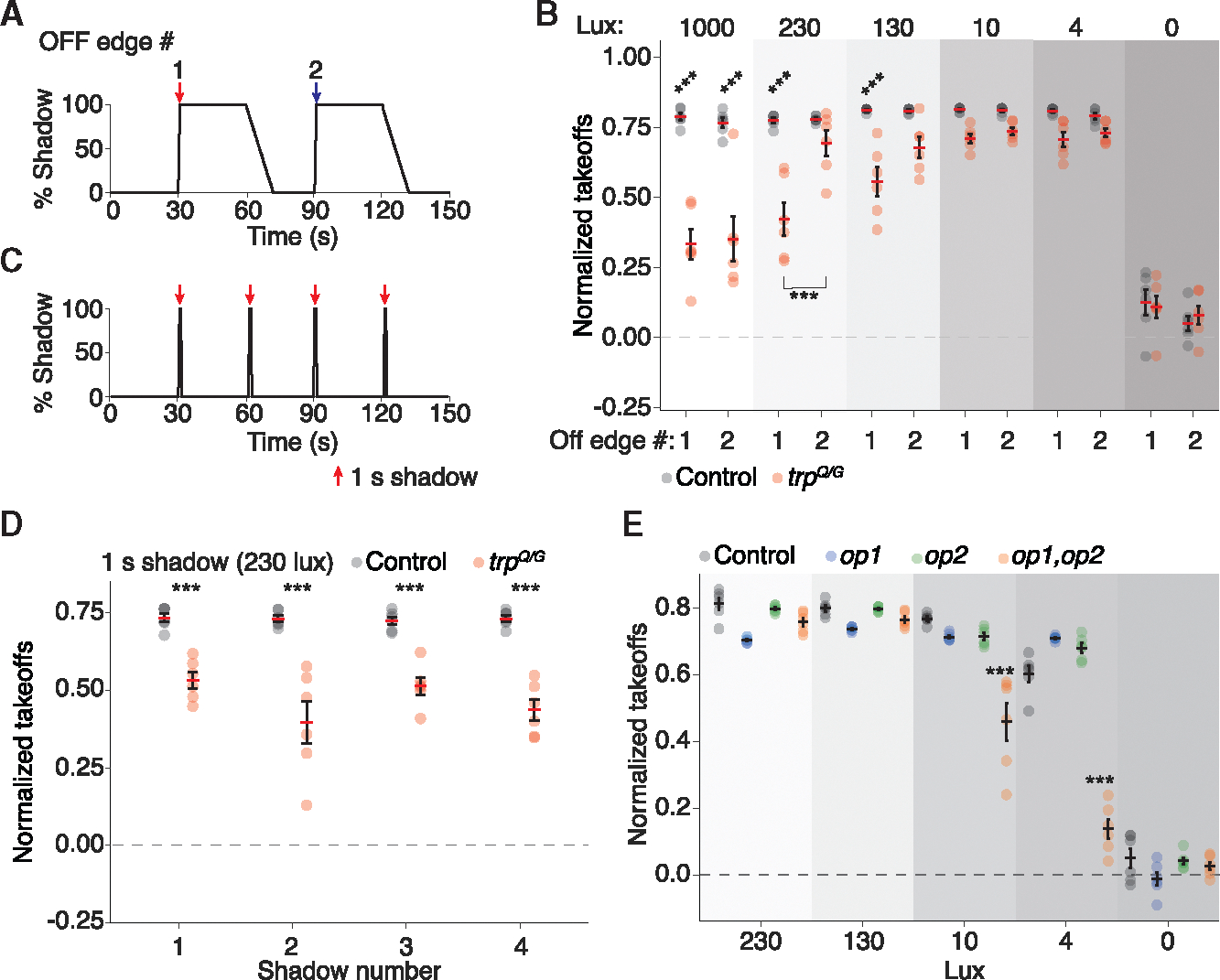
TRP and opsins impact shadow detection at different light intensities (A) Shadow-movement pattern with 30-s dark time between the OFF- and ON-edge shadows. The initiation of the first (1) and second (2) OFF-edge shadows are indicated. The first OFF-edge shadow was preceded by ≥7 min of light. The second OFF-edge shadow was preceded by 30 s in the dark and then 30 s of light (11 s of ON-edge shadow and 18 s of full light). (B) Normalized takeoffs from control and *trp*^*Q/G*^ mosquitoes exposed to the two OFF-edge shadows indicated in (A).Three-way ANOVA with factors of genotype (*p* < 0.001), dark time (*p* < 0.05), and light intensity (*p* < 0.001). Group means compared by Tukey’s honestly significant difference (HSD) test. (C) One-second dark time between each of the four OFF- and ON-edge shadows. (D) Normalized takeoffs from control and *trp*^*Q/G*^ mosquitoes under the 1-second-long dark time shadow protocol indicated in (C). Two-way ANOVA with factors of genotype (*p* < 0.001) and shadow-movement number (*p* = 0.12). Group means were compared using Tukey’s HSD test. (E) Normalized takeoffs by the indicated mosquitoes at 4–230 lux or in the dark (0 lux). Two-way ANOVA with factors of genotype (*p* < 0.001), lux (*p* < 0.001), and genotype:lux interaction (*p* < 0.001). *n* = 6 cages of 30 female mosquitoes per condition, each recorded three times. ***p < 0.001. *p < 0.05. Means ± SEMs. See also Figures S5 and S6.

To analyze the shadow response exhibited by *trp*^*Q/G*^, we used 5-day-old females since the amplitude of the light response was significantly reduced in these mosquitoes, and because 1-day-old control females do not exhibit robust host-seeking behavior. We exposed the females to 230 lux, and two 60 cm/s OFF-edge shadows at 30 and 90 s (Figure 7A). In response to the first OFF-edge shadow (at 30 s), the *trp*^*Q/G*^ mutants were significantly less responsive to a fast-moving shadow than the wild-type control (Figure 7B; normalized takeoffs: control, 0.78 ± 0.01; *trp*^*Q/G*^, 0.42 ± 0.06). Surprisingly, in response to the second OFF-edge shadow (at 90 s), the control and *trp*^*Q/G*^ mutants exhibited similar aversive responses (Figure 7B; normalized takeoffs: control, 0.78 ± 0.01; *trp*^*Q/G*^, 0.72 ± 0.02). This was unexpected since the only difference between the first and second OFF-edge shadows is that the first OFF-edge shadow was preceded by light for >7 min, while the second OFF-edge shadow was exposed to light for only ~30 s (15 s during the ON-edge shadow and 15 s of 230 lux).

Given the effects of light on the behavioral aversion by the *trp*^*Q/G*^ mutant in response to the first and second OFF-edge shadows, we tested whether the differences would be reduced at lower light intensities. *trp*^*Q/G*^ was still less responsive to the first exposure to 130-lux light (Figure 7B). Strikingly, the effect of prior light exposure was largely lost when *trp*^*Q/G*^ mosquitoes were housed under 10- or 4-lux light, and they had nearly equivalent takeoff rates upon exposure to the first and second OFF-edge shadow, which was similar to the control (Figure 7B). Thus, the requirement for *trp* for avoiding a threatening shadow is limited to higher light levels. To test the impact of light intensity further, we exposed the mosquitoes to shadows in the presence of 1,000-lux light. At this intensity, the *trp*^*Q/G*^ aversion to the first and second OFF-edge shadows was severely impaired (Figure 7B).

To confirm that the increase in the *trp* mutant response to a moving shadow was the result of prior light exposure, rather than an effect from successive moving shadows, we implemented a second shadow-movement protocol (Figure 7C). We exposed the mosquitoes to four 60-cm/s shadows (every 30 s). Both the OFF- and ON-edge-shadows were rapid so that the mosquitoes experienced only 1 s in the dark between each OFF-edge and ON-edge shadow. Control mosquitoes tested with this protocol displayed a normalized takeoff rate of ~0.74, whereas the takeoff rate of the *trp*^*Q/G*^ was significantly lower during each shadow movement (Figure 7D). This indicates that the *trp* mutants were not especially sensitive to repeated aversive visual stimuli. Since the mosquitoes were not exposed to a long dark adaptation, these results demonstrate that dark adaptation is necessary to suppress the visual defects preventing *trp*^*Q/G*^ from exhibiting a robust aversive response to a threatening shadow. Though the participation rate of the *trp*^*Q/G*^ mutant was lower than the control (17.6 ± 0.7 in control vs. 12.2 ± 0.3 in *trp*), participation rate and shadow response were uncorrelated (Figures S6A and S6B).

We performed additional ERG recordings on the *trp* mutant, so that their exposure to light roughly mimicked that during the behavioral recordings. We first exposed the mutants to 7 min of 230-lux light, then dark-adapted them for 1 or 30 s, after which we re-exposed them to 230 lux, and performed ERGs (Figure S5C). In controls, 1 or 30 s of dark adaptation prior to the second light stimulus resulted in ERG responses of 3.61 ± 0.55 mV and 5.30 ± 0.29 mV, respectively (Figures S5C–S5E). This represented an ~45% increase in amplitude after the longer dark adaptation. In the case of the *trp* mutant, there was a much larger response after 30 s of dark adaptation, as the ERG amplitude was nearly flat following 1 s in the dark and increased >6-fold with 30 s of dark adaptation (Figures S5C–S5E; 0.45 ± 0.15 mV to 2.81 ± 0.65 mV). Consistent with the behavioral data, these results indicate that 30 s of dark adaptation is necessary for *trp*^*Q/G*^ to partially recover its sensitivity to 230-lux light.

### Opsins 1 and 2 required for detecting visual threats under low-light intensities

Given that TRP contributes to shadow avoidance only at the higher light intensities, we tested whether disrupting a subset of the light sensors, rhodopsins, would also impair shadow avoidance at specific light levels. *Ae. aegypti* expresses five rhodopsins in its compound eyes. The major opsin, Op1, is expressed in the majority of the photoreceptor cells—the six outer photoreceptor cells (R1–6) and many of the centrally located R8 cells.^21^ Nevertheless, mutation of *op1* does not cause an obvious defect in the ERG, or disruption of light-dependent behaviors.^22^ Similarly, elimination of Op2, which is the opsin most similar to Op1 (~90% identical) and is expressed in a subset of R7 photoreceptor cells,^23^ also does not cause discernible effects on light-driven behaviors, or on the ERG.^22^ Eliminating both Op1 and Op2 impairs the mosquito’s ability to visually detect hosts^22^ and navigate toward attractive wave-lengths of light.^24^ Nevertheless, *op1*,*op2* mutants maintain an ERG response to light, although it is reduced in amplitude.^22^

To test whether the *op1* and *op2* single mutants, and the *op1*,*op2* double mutant impair the detection of visual threats, we subjected the mosquitoes to 60 cm/s OFF-edge shadows under 230to 0-lux light. The control, *op1*, and *op2* mosquitoes showed a similar participation rate in our assay (Figure S6C; average landed mosquitoes: control, 16.5 ± 0.6; *op1*^*2*^, 17.2 ± 0.4; *op2*^*1*^, 15.4 ± 0.5), whereas the *op1*^*2*^,*op2*^*1*^ double mutant displayed a slightly elevated participation (Figure S6C; 18.9 ± 0.4 average landed mosquitoes). Mutations in either *op1*^*2*^ or *op2*^*1*^ did not significantly reduce the takeoff rate (Figure 7E). Similarly, the *op1*^*2*^,*op2*^*1*^ double mutant was also highly responsive to fast-moving shadows at 230 and 130 lux (Figure 7E). However, the takeoffs exhibited by the double mutant fell significantly at 10 and 4 lux (Figure 7E). This effect is unlikely to reflect differences in participation, as the participation rate minimally predicted takeoffs in response to a moving shadow (Figure S6D). These data demonstrate that Op1 and Op2 together contribute to the sensitivity of the eye to OFF-edge shadows at low-light intensities only.

## DISCUSSION

### Drive to host seek is temporarily interrupted by a visual threat

Reproduction in female *Ae. aegypti* relies on acquiring a blood meal. This endeavor is risky, as mammalian targets defend themselves against being bitten. While humans often use their hand or wield a swatter to attack a mosquito, other mammals employ different strategies, such as swishing their tails back and forth as a deterrent.^10^ These approaches present mosquitoes with aversive visual stimuli that it must respond to. Here, we presented host-seeking mosquitoes with a visual cue simulating a threat: a fast-moving shadow. Unlike previous studies that used a mechanical swatter to strike at flying mosquitoes,^7,8^ our stimulus was purely visual, and applied to mosquitoes that were landed and actively host seeking. In contrast to a “looming stimulus,” which expands in size, simulating an approaching predator,^9^ the edge size of our stimulus was fixed. We observed that a moving shadow strongly triggered an escape response, but only when the shadow moved at high speeds and created an OFF-edge. This demonstrates that mosquitoes use moving edges to assess the danger posed by objects in their environment.

We found that female mosquitoes transiently abandon host seeking in response to an imminent visual threat. This lapse in host seeking is only momentary. The mosquitoes commonly re-landed after a threat-induced takeoff, consistent with a previous observation.^9^ Unlike females, males do not draw blood meals, and in our assays do not host seek. Like females, males readily respond to a shadow-mediated visual threat, albeit at a lower level than females. It is plausible that the minor sexual dimorphism to a visual threat in *Aedes* is because only females host seek and are therefore particularly attuned to danger in their environment.

### Motion vision vs. illuminance detection in the *Aedes* threat response

The shadow stimulus in our assay has two primary optical properties, either of which could account for mosquitoes’ response to it. First, the stimulus presented a fast-moving edge as the shadow traversed across the arena. Second, the stimulus rapidly changed the total illuminance in the arena as the light blocker occluded (OFF-edge shadow) or revealed (ON-edge shadow) the light source. To determine which of these visual properties predominated in stimulating evasive behaviors, we compared the response to a flashing light vs. the response from a moving shadow. Flashing the lights off (i.e., changing total illuminance without casting a shadow) was less than half as aversive as a moving shadow, indicating that the response to the shadow was primarily from its moving edge. We also determined that a fast-moving OFF-edge shadow was more aversive than a fast-moving ON-edge shadow. The response to an ON-edge shadow was roughly equivalent to their light flash off response. It is unlikely that takeoffs resulting from an ON-edge are simply the result of changes to total illuminance, given that the ON-edge response was substantially higher than the response to a light flash. This suggests that mosquitoes are detecting both ON- and OFF-edge shadows, but that an OFF-edge is significantly more aversive, perhaps because it is accompanied by a decrease in illuminance.

In *Drosophila*, distinct neurons in the lamina (the first neuropile of the optic lobe) and the lobula plate (the last neuropile of the optic lobe) discriminate between ON and OFF edges.^25–27^ The optic lobes of *Ae. aegypti* are anatomically similar to *Drosophila*, and also contain motion-responsive neurons in the lobula.^28,29^ Currently, the accounting of how the *Aedes* visual system processes edges is far from complete. Here, we provide the first report that ON and OFF edges yield significantly different behavioral outputs in *Aedes*, as OFF edges are substantially more aversive. The T5 cells in *Drosophila*, which respond specifically to OFF edges, are tuned to detect higher-velocity movement than T4 cells, which respond to ON edges.^30^ The teleological explanation for this could be that from the perspective of a fruit fly or mosquito, an inbound predator is more likely to obstruct light, producing an OFF-edge, than to reveal light, producing an ON-edge.

### Differential effects of phototransduction proteins on visual threat detection

To examine how photoreceptor cell inputs affect the ability of *Ae. aegypti* to detect a moving visual threat, we examined three separate phototransduction mutants: *op1*, *op2*, and *trp*. Interestingly, we observed differential effects from these various mutants. The *Aedes trp* mutant is unable to detect a shadow, but only at a high light intensity. In contrast, *Ae. aegypti* harboring null mutations in either *op1* or *op2* behave like wild-type in terms of their response to a moving shadow, even under low-light conditions. This is remarkable given that Op1 is the major opsin in *Aedes* and is expressed in all R1–6 photoreceptor cells and many R8 cells.^21^ In *Drosophila*, a functionally equivalent opsin is Rh1, which is also expressed in the R1–6 cells.^31^ Mutating *Drosophila rh1* dramatically impairs motion vision.^32,33^ In contrast, we found that the *Aedes op1* mutant readily detects a moving shadow, and we showed previously that it retains an optomotor response (a measure of the ability to perceive optic flow), although it is reduced relative to wild-type.^22^ This indicates one of two possibilities. First, it is possible that there is an unknown light sensor that is also expressed in *Aedes* R1–6 cells; however, this is unlikely. Rather, we support a second possibility that unlike *Drosophila,*^32^ the R7 and R8 cells in *Aedes* significantly contribute to motion vision.

### Remarkable light sensitivity of *Aedes aegypti*

A distinguishing feature of *Aedes*’ compound eyes is that their rhabdomeres have a closed configuration such that each abuts its neighbor, forming a singular ring within the ommatidium.^11^ In contrast, *Drosophila* rhabdomeres exist in an open configuration, so that none of the rhabdomeres are in contact with each other. A potential benefit of *Aedes*’ fused or closed rhabdom is that it likely enhances photon capture under low-light conditions. A drawback is that this light sensitivity comes at the expense of visual acuity. By contrast, the open rhabdomere allows for greater spatial resolution due to neural superposition—i.e., rhabdomeres in different ommatidia, each with a parallel optical axis, receive light from the same position in space, and signal to the same downstream neuron.^34,35^ Nevertheless, the closed rhabdom in *Ae. aegypti* likely accounts for their ability to sense threatening visual cues under very dim ambient light conditions.

### Limitations of the study

We demonstrate that host-seeking *Ae. aegypti* can sense a moving edge from a shadow, and that this triggers a robust escape response. Although the escape response from a threatening shadow was robust even under exquisitely low-light conditions, due to technical limitations, we were unable to determine the lower limit of this sensitivity, since we could not test intensities less than 4 lux. Additionally, we only tested one shadow shape—a single moving edge. It is plausible that other shapes, such as those more closely resembling a mosquito predator, would have maintained their aversiveness even when moving at a very slow speed.

We found that the mosquito’s aversion to a shadow is strongest when the shadow decreases rather than increases overall illuminance. The ability to detect this stimulus relies on functional photoreceptor cells, as loss of both Op1 and Op2, or TRP, impairs shadow detection, but at distinct light levels. Although Op1 is the major opsin in *Aedes*, we did not observe a defect in shadow avoidance in *op1* mutants. Instead, only mosquitoes that were doubly mutant for *op1* and *op2* exhibited deficits in shadow detection, but only under low light. Op1 is expressed in R1–6 photoreceptors as well as most R8 cells,^21^ whereas Op2 is expressed in some R7 cells.^23^ Therefore, eliminating both Op1 and Op2 would render some of the mosquito’s ommatidia completely insensitive to light. This effect may be particularly damaging to motion vision, which, in other dipterans, relies on the sequential activation of photoreceptor cells in adjacent ommatidium in response to a moving edge.^25^ Although we have not directly tested this, eliminating the inputs from an entire ommatidia may disrupt the paired photoreceptor activity that is required in this pathway. In addition to Op2, two other opsins, Op8 and Op10, are also expressed in R7 cells.^36^ Therefore, we suggest that *op8*,*op10*;*op1* triple mutants might have similar defects in shadow detection. At present, the genetic reagents for testing this idea are unavailable.

Our characterization of *Aedes* TRP in phototransduction also yielded unexpected results that, at present, we cannot mechanistically explain. Unlike the *Drosophila trp* mutant, the photoreceptor cells in the *Aedes trp* mutant never fully lost the maintained component of the ERG under high-intensity light (1,000 lux). Loss of the ERG response in *Drosophila trp* mutants is from PIP_2_ depletion from the photoreceptor cell membrane, as an influx of Ca^2+^ from the TRP channel inactivates the phospholipase encoded by the *norpA* gene.^37^ Conceptually, we do not understand how the *Aedes trp* mutant sustains its ERG response, albeit diminished relative to wild-type. We propose several non-mutually exclusive possibilities. First, it is possible that another cation channel, such as *Aedes* TRPL, contributes sufficient Ca^2+^ during the light response to diminish NORPA activity. Second, *Aedes* NORPA may require less Ca^2+^ than *Drosophila* NORPA for inactivation. Third, *Aedes* may have a substantially faster rate of PIP_2_ regeneration compared with *Drosophila*. Consequently, PIP_2_ is never fully depleted.

Although our work identified some of the genes required in photoreceptor cells for motion vision in *Aedes*, the photoreceptor cells are only capable of responding to illuminance, and on their own do not encode motion. At this time, we do not know the projection neurons in the *Aedes* optic lobes that synapse with the photoreceptor cells and function in the circuits that underlie motion vision. We speculate that there will be notable distinctions between the ONand OFF-edge pathways, given the differences in behavior produced by these two stimuli.

## RESOURCE AVAILABILITY

### Lead contact

Further information and requests for resources should be directed to the lead contact, Craig Montell (cmontell@ucsb.edu).

### Materials availability

Requests for reagents generated in this work may be directed to the lead contact, Craig Montell (cmontell@ucsb.edu).

### Data and code availability

- Mosquito tracking code is available on GitHub and Zenodo, https://doi.org/10.5281/zenodo.14826855.
- All raw data and videos are available on Dryad: https://doi.org/10.5061/dryad.j6q573nr3.
- Any additional information required to reanalyze the data reported in this paper is available from the lead contact upon request.

## STAR★METHODS

### EXPERIMENTAL MODEL AND STUDY PARTICIPANT DETAILS

The wild-type control mosquitoes used in behavioral and imaging experiments were *Ae. aegypti* (Liverpool strain), reared at 28°C and 80% relative humidity under a 14:10 light:dark cycle in walk-in chambers. Eggs were hatched in deionized water and fed fish food (TetraMin tropical granules, Tetra) until pupation. Pupae were then placed into an insect collection cage (17.5 × 17.5 × 17.5 cm, BugDorm-4S1515) for maintenance. Adult mosquitoes were fed 10% sucrose in glass bottles with cotton wicks. In order to collect eggs, adult females (≥5 days and < ~30 days) were fed defibrinated sheep blood (Hemostat, <7 days old) using an artificial feeder (Hemotek). All mutant mosquitoes were outcrossed to the Liverpool strain of *Ae. aegypti* for ≥5 generations.

### METHOD DETAILS

#### Behavioral cages and experimental conditions

The cages used for behavioral experiments were insect collection cages (BugDorm-4S1515) modified by replacing up to three of the mesh walls with clear acrylic (McMaster #8560K171) for video recording. Cages were placed in a temperature-controlled incubator set to 28°C, with the clear acrylic side oriented toward the camera and a mesh oriented toward the IR light source. For experiments involving female *Ae. aegypti*, each cage was loaded with 30 mated, non-blood-fed adults (5–21-days old) via a mouth pipette. Before performing the assays, the mosquitoes were allowed to acclimatize to the cage for ≥12 h in the presence of 10% sucrose, which served as a food source. An identical protocol was followed for behavioral experiments performed on males, except that cages were loaded with 45 mosquitoes. All assays were performed at either ZT 0 or ZT 10. Immediately prior to the behavioral recordings, sucrose bottles were removed from the behavioral cages. Human odor from a worn glove was applied to the back wall of the cage before each recording, and CO_2_ (from human breath) was blown briefly into the cage. The cage was then placed in a temperature-controlled incubator (28°C, 80% humidity) with a mesh side pushed against the IR light diffuser (Figure 1A), which warmed the back of the cage to 33°C. For experiments involving sugar attraction, we used a sucrose-soaked paper towel to apply a sucrose solution to the back of the cage. For sucrose attraction experiments involving females, we did not apply human odor to the back of the cage and blocked heat from our IR lights by adding an additional 3-mm-thick piece of polycarbonate plastic in front of the lights.

#### Shadow threat assay and video acquisition

To cast a moving shadow on the mosquitoes, we placed a 22-cm-long belt-driven moveable stage 33 cm in front of the behavioral cage. Attached to the stage was a custom-fabricated mount that secured a 13.5 cm × 6 cm × 1.9 mm clear plexiglass window. On top of this window, we placed a rectangular strip of white, opaque electrical tape (3 cm × 6 cm), which when moved in front of our white LED light source (Figure S1C; Outerbanks Provisions) blocked visible light (Figures 1B and 1C). Movement of the stage was achieved by a Nema-17 stepper motor, which was wired to an A4988 motor driver (StepperOnline), set to 4V using the builtin potentiometer. The motor driver received movement commands from an Arduino Uno (Egloo), and the speed of the stage was modified by changing the pause length between each motor step. The mosquitoes were recorded with a varifocal webcam (ELP), affixed with an IR pass filter (Heliopan), at 30 frames per second, and at a resolution of 640 × 480 pixels. To provide constant illumination for the experiments, we placed near IR (850 nm) LED lights (Waveform) behind our behavioral cage. The IR lights were shown through a translucent white acrylic panel (30.5 cm × 30.5 cm × 3 mm), which served as a light diffuser.

A custom Python (version 3.11) script acquired video frames (using OpenCV library) and sent commands to the Arduino Uno (using pySerial library), which moved the light blocker to a given position, at a particular time, and at a particular speed. This enabled us to generate moving shadows that traveled up to 60 cm/s. Mosquitoes were exposed to a moving shadow every 30 s over the span of a 150 s recording, unless otherwise indicated. The Arduino was programmed to receive commands sent from our Python script to the serial port, which set the number of motor steps, the direction of movement, and the pause length between each step, which served to modify the speed of the light blocker. The script paired each video frame to a timestamp as well as to the stage position. This allowed us to monitor mosquito behavior as our experiment progressed and as the light blocker was moved. The ability to pair stage positions to video frames enabled us to calibrate our assay, matching each stage position to the shadow it cast on the back wall of the arena (Figure S1D).

#### Light recordings

To record the spectrum of the light source (Figure S1C), we used a ThorLabs CCS100 compact spectrometer. Lux measurements were made with an LT40 LED light meter (Extech). Light power measurements were made using a ThorLabs PM100D compact power and energy meter console, coupled to a fiber optic cable.

#### Video tracking of mosquitoes

We analyzed each behavioral recording using custom 2D video-tracking code written in MATLAB (version 2021a). These scripts function in two successive steps that can be classified into two categories: those that identify foreground objects (i.e., landed mosquitoes) and those that match individual mosquitoes from frame to frame (Figures S1A and S1B).

For detecting the position of female mosquitoes along the back wall of our cage, we leveraged the fact that they are frequently moving and taking off. Namely, we created a unique background model that corresponded to every 30-s interval in the videos. This background model was the median pixel value from 100 randomly-drawn grayscale frames (Figure S1A, Background model). We then took the absolute difference between the background model and a given frame, which yielded an array wherein high magnitude values corresponded to moving pixels. To separate partially overlapping mosquitoes, we convolved this frame with a Laplacian of Gaussian spatial filter. We then thresholded the resulting image by setting pixels whose value fell in the top 40^th^ percentile to 1, and those in the lower 60^th^ percentile to zero. Lastly, from this binary frame, we stored the centroid coordinates of blobs with an area between 20 and 60 pixels.^2^

For experiments involving male mosquitoes, we used a nearly identical approach. Because male mosquitoes are largely stationary, we omitted the use of a median-pixel background model. Instead, we used a 2D bandpass filter to identify regions of dark pixels (male mosquitoes) in the foreground of each frame. This approach yielded comparable results to our female-mosquito-tracking code but was considerably slower and less resilient to debris in the foreground, and therefore was only used in experiments involving males.

After identifying the centroid positions of mosquitoes for every frame in a video, we implemented the following matching algorithm to follow mosquitoes in consecutive frames (Figure S1B). The positions of the first-detected mosquitoes were assigned as tracked mosquitoes (Figure S1B, ‘start’ and ‘1’). Then, for subsequent frames, new detections were matched to this set. Specifically, for each time step (1 frame, ~33 ms), we used a 2D constant-velocity Kalman Filter to predict the new location of each mosquito. This allowed us to predict the location of mosquitoes whose position along the back wall was occluded, most often by another mosquito transiently flying over its location. We then matched the Kalman-predicted position of each mosquito to the set of detections corresponding to the video frame using the Hungarian Algorithm. Only matches with a Euclidean distance of <10 pixels were considered as a positive match (Figure S1B, and ‘2’). This distance threshold largely eliminated the matching of flying mosquitoes, whose velocity almost always exceeded 10 pixels/frame. For positive matches, we then updated the location of the mosquito track (Figure S1B, and ‘3’). Unmatched detections were assigned a new mosquito track ID, indicating that a new mosquito had landed on the back wall of the cage. Unmatched mosquito tracks were marked as being invisible (Figure S1B, and ‘4’). Mosquito tracks invisible for >6 consecutive frames were deemed to be lost (i.e., no longer considered for matching detections) and later considered as a possible takeoff event. This process was then repeated for each frame in a video.

#### Classifying searching behavior

Only mosquito tracks visible for ≥30 frames (≥1 s) were considered for behavioral analysis. We calculated searching behavior by examining the walking speed of mosquitoes on the back wall of the cage. Specifically, we classified mosquitoes as searching when their 1-s-long moving-median speed was between 6 and 52 pixels/second. The upper bound of this classification helped to avoid misclassifying mosquitoes initiating a takeoff as searching, as we failed to observe walking that exceeded 52 pixels/second. Searching bouts that began within 1 s of each other (two bouts separated by < 30 frames) were scored as being one contiguous searching bout.

#### Calculating behavior transition probabilities

We calculated the behavior transition probabilities shown in Figure 3 from four separate experiments, each comprising 18 videos from 6 cages of 30 female mosquitoes. Together, these data contained >6,500 mosquito tracks generated from ~700 mosquitoes. To calculate the average probability of a behavioral transition, we randomly sampled 50,000 2-s bins (~60 continuous frames) from each experiment (group of 18 videos). A behavioral transition between being stationary and searching was scored when a bin began with one behavior and then switched to another for >50% of the frames. For example, a sample where a mosquito was stationary for the first 5 frames then switched to searching for the remaining 55 frames would be scored as a stationary-to-searching switch. If at any point in the sample a mosquito took off, its behavior was scored as switching to takeoff.

#### Examining morphology of ommatidia using TEM

For eye imaging via transmission electron microscopy, we dissected retinas from 5- and 10-day old male and female control (Liverpool) and *trp*^*Q/G*^ mosquitoes (*n* = 3 for each age and genotype) and fixed them overnight at 4°C in a 0.1 M phosphate buffer (pH 7.2) solution with 2% glutaraldehyde. The following day, we incubated the samples in a solution of 0.1 M phosphate buffer (pH 7.2) and 1% osmium tetroxide for ≥2 h. After fixation, the samples were gradually dehydrated in a solution of ethanol from 25% to 100%. Samples were then infiltrated with epoxy resin (Electron Microscopy Sciences, 14310), embedded in polyethylene molding trays, and cured in an oven overnight at 60°C. Once the resin was hardened, a glass knife on a Reichert-Jung Ultracut microtome was used to generate 90 nm ultra-thin sections from the distal regions of the retinas. The sections were then stained with toluidine blue and examined under a light microscope to ensure proper orientation and thickness. Samples were then placed onto Formvar London Finder Grids (CU, LF135). The samples were visualized using a Thermo Scientific Talos F200X transmission electron microscope.

#### Generation of *trp* mutant by CRISPR-Cas9

The two *trp* alleles (*trp*^*QF2*^ and *trp*^*GFP*^) were generated by CRISPR-mediated homology-directed repair (HDR). We used the CRISPR Optimal Target Finder (https://flycrispr.org/target-finder/) to choose short-guide RNAs (sgRNAs) that targeted the *trp* locus (LOC5566497). Each of these alleles included a fluorescent reporter expressed in the eye under control of the *3xP3* promoter to allow identification of transgenic mosquitoes (dsRed for *trp*^*QF2*^ and GFP for *trp*^*GFP*^). The *trp*^*QF2*^ allele included the sequences encoding a QF2 reporter flanked by the T2A self-cleavage peptide and a dsRed marker, which interrupted the DNA region coding for residue 335 just before transmembrane domain one. The *trp*^*GFP*^ allele included an insertion of the sequence encoding a GFP reporter, which disrupted the codon for residue 447 of transmembrane three. The target sequences of the sgRNAs fragments were synthesized by gBlocks Gene Fragments (IDT). The sgRNA sequences are given in Table S1.

To generate the *trp*^*QF2*^ and *trp*^*GFP*^ constructs, we inserted sgRNA sequences into the previously-generated plasmids pAaU6-sgRNAscaffold-T2A::QF2–3xP3-DsRed and pAaU6-sgRNAscaffold-3xP3-GFP.^23^ This was achieved by using KpnI and SpeI restriction sites, and the insertion was facilitated by the In-Fusion cloning kit (Clontech). For the *trp*^*QF2*^ construct, we inserted a 948 bp upstream homology arm into the pAaU6-sgRNA-T2A::QF2–3xP3-DsRed plasmid. This was done using the AvrII restriction site, placing it in-frame with the *T2A::QF2* sequence at the *trp* locus, which was followed by the insertion of a *3xP3-DsRed* fragment. A 809 bp downstream homology arm was then inserted using the AfeI restriction site, resulting in the pAaU6-*trp*^*QF2*^-T2A::QF2-gRNA-arm1–3xP3-DsRed-arm2 plasmid. For the *trp*^*GFP*^ allele, we inserted a 1041 bp upstream homology arm into the pAaU6-sgRNA-3xP3-GFP plasmid using the NheI restriction site, followed by a *3xP3-GFP* fragment. A 1041 bp downstream homology arm was then inserted using the PacI restriction site, generating the pAaU6-trp^GFP^-gRNA-arm1–3xP3-GFP-arm2 plasmid. Homology arms for both alleles were generated by PCR amplification of genomic DNA extracted from the Liverpool strain. Additionally, each plasmid contained the SV40 transcription terminator, the U6 promoter for directing sgRNA expression, and the sgRNA scaffold.^22^ The plasmid sequences were verified by DNA sequencing. The primers used to generate the upstream and downstream homology arms for *trp*^*GFP*^ and *trp*^*QF2*^ are listed in Table S1.

To generate the *trp*-mutant mosquitoes, we microinjected the *trp*^*QF2*^ and *trp*^*GFP*^ plasmids into embryos of mosquitoes expressing Cas9 under control of the *ubiquitin L40* promotor (gift from O.S. Akbari). We collected freshly laid embryos, and microinjected the plasmid DNA (500 ng/mL) using a quartz needle (quartz glass capillaries; OD, 1.0 mm; ID,0.7 mm; length, 10 cm; Sutter Instrument, cat. #Q100–70-10, pulled by a micropipette puller, Sutter Instrument, p-2000) into the posterior ends of ~2,000 embryos using a micro-injector (Eppendorf) and a Zeiss Axioplan 2 microscope to visualize the embryos. G_0_ embryos hatched four days post-injection, and the adult G_0_ animals (~100 per injection) were crossed to the opposite sex. The females were then blood-fed to generate G_1_ progeny. These mosquitoes were then screened on the basis of a fluorescent eye marker, dsRed or GFP, under a Zeiss SteREO Discovery V8 stereomicroscope. Positive G_1_ animals were genotyped by PCR and out-crossed to the wild-type control strain (Liverpool) for ≥5 generations. Primers used for genotyping are listed in Table S1.

#### Quantitative PCR (RT-qPCR)

To determine whether we could detect *trp* mRNA in the transheterozygous mosquitoes (*trp*^*Q/G*^), we extracted RNA from 5 heads per sample with TRIzol reagent (Thermo Fisher Scientific). We used 1 μg RNA for cDNA synthesis (Promega). The RT-qPCR reactions were performed using LightCycler 480 SYBR Green I Master Mix (Roche). We measured the relative expression of mRNA expression of *trp* via the ΔΔCT method using *Rps7* (LOC5572090) expression as an internal control. Primers used for RT-qPCR are listed in Table S1.

#### Electroretinogram recordings

ERG recordings were performed in a manner similar to what we described previously.^22^ Briefly, each electrode was placed in a glass needle (Thin - wallglass capillaries World - TW110F-3) that was prepared using a micropipette puller (Sutter Instruments- Model P-97), and filled with Ringer’s solution (3 mM CaCl_2_, 182 mM KCl, 46 mM NaCl, 10 mM Tris pH 7.2). Mosquitoes were secured to glass coverslips using melted beeswax. The electrodes were applied to small drops of electrode cream (Parker) on one eye (for the recording electrode) and on the thorax (for the reference electrode). To perform the ERGs, the mosquitoes were exposed to white light (Apex illuminator) directed through a light guide. The signals were amplified using an IE-210 amplifier (Warner Instruments) coupled to a Powerlab 4/30 device (AD Instruments). The raw recordings were sampled at 2000 Hz. For the purposes of plotting the ERG response from multiple mosquitoes, we averaged the raw data in 100 ms bins. To determine the final amplitude of each ERG response before terminating the light stimuli (Figures 6P and 6Q), we recorded the voltage coincident with cessation of the light stimulus, and subtracted that value from the highest voltage we observed up to 2 s after the light stimulus had ended.

#### Confocal imaging using the *trp QF2* reporter

To determine the expression pattern of the *trp QF2* reporter, we crossed virgin female *trp*^*QF2*^ to *QUAS-mCD8::GFP*^38^ males and imaged the adult females. We fixed the heads of 7–10-day-old mosquitoes for 12 h at 4°C in 4% paraformaldehyde in PBST, which consisted of PBS (137 mM NaCl, 2.7 mM KCl, 8 mM Na2HPO4, 2 mM KH2PO4) and 0.3% Triton X-100. We then dissected retinas from females, and washed them 3 times for 15 min in 0.3% PBST. Samples were then incubated with Alexa Fluor 633 phalloidin (#A22284) for staining actin, which primarily labeled the rhabdomeres. No primary or secondary antibodies were used to visualize GFP expression. The samples were mounted in Vectashield (Vector Laboratory, H-1000) and imaged using a Zeiss Inverted LSM 900 laser scanning confocal microscope using a 40x/1.3 DIC oil immersion-corrected objective. Immersol 518F was used as the immersion medium to match the refractive index of the Vectashield mounting medium. Images were acquired with 14 z-slices at a slice depth of 1 μm at a resolution of 1024 × 1024 pixels. To visualize the staining, we used ImageJ software to generate a max-intensity projection that spanned the proximal and distal layer of the retina. Shown in Figure 6C is an image from an optical section at a depth of 13 μm.

#### Genotyping *op1*^2^ and *op2*^*1*^ mosquitoes

Primers used to genotype the *op1*^*2*^ and *op2*^*1*^ mosquitoes are listed in Table S1.

### QUANTIFICATION AND STATISTICAL ANALYSIS

All behavioral experiments were performed on six cages of mosquitoes. For experiments involving female *Aedes*, each contained 30 insects. Experiments performed on males contained 45 mosquitoes. Reported are the mean values from three technical replicates per cage. Exact ‘n’s can be found in the figure legends corresponding to each figure. On average, technical replicates of the same cage were separated by 22 ± 2 min. For analyzing takeoffs, we excluded replicates where <10 mosquitoes were landed on the back wall prior to the stimulus. All ERG recordings were performed on ≥6 mosquitoes. To calculate the participation rate, we calculated the mean number of mosquitoes that were landed on the back wall of the cage over the span of the recording. Precise information regarding the statistical tests used in each panel can be found in the corresponding figure legends. Briefly, for experiments with a full factorial design, we used one-, two-, or three-way ANOVA to examine the effect of various factors on mosquito behavior, as well as the interaction effect from these factors. For experiments with an incomplete factorial design, we used one-way ANOVA with a single factor comprising all possible level combinations of the original factors. We used Tukey’s Honest Significant Difference (HSD) test post hoc to compare group means. *p* values were calculated using R (R Studio, v.1.4.1717).

## Supplementary Material

1

2

3

4

5

## Figures and Tables

**KEY RESOURCES TABLE:** 

REAGENT or RESOURCE	SOURCE	IDENTIFIER
Chemicals, peptides, and recombinant proteins		
Alexa Fluor 633 phalloidin	Thermo Fisher Scientific	A22284
Epoxy resin	Electron Microscopy Sciences	14310
Formvar London Finder Grid	Electron Microscopy Sciences	CU, L135
TRIzol	Thermo Fisher Scientific	15596026
Electrode cream	Parker Laboratories	PM-017-0001C
Vectashield	Vector Laboratory	H-1000
Critical commercial assays		
In-Fusion cloning kit	Takara Biosciences	638909
cDNA synthesis kit	Promega	A5003
LightCycler 480 SYBR Green Master	Roche	04707516001
Deposited data		
Data	This paper	https://doi.org/10.5061/dryad.j6q573nr3
Code	This paper	https://doi.org/10.5281/zenodo.14826855
Experimental models: Organisms/strains		
*Ae. aegypti*: wild type	Akbari Lab	N/A
*Ae. aegypti*: ubiquitin L40 promoter Cas9	Akbari Lab	N/A
*Ae. aegypti: trp^QF2^*	This paper	N/A
*Ae. aegypti: trp^GFP^*	This paper	N/A
*Ae. aegypti: op1^2^*	Zhan et al.^22^	N/A
*Ae. aegypti: op2^1^*	Zhan et al.^22^	N/A
*Ae. aegypti:* QUAS-mCD8:GFP	Wang et al.^38^	N/A
Oligonucleotides		
sgRNA sequences	This paper	Table S1
Genotyping primers	This paper	Table S1
RT-qPCR primers	This paper	Table S1
Recombinant DNA		
*trp-3xP3-GFP-HDR*	This paper	N/A
*trp-3xP3-DsRed-QF2-HDR*	This paper	N/A
Software and algorithms		
Python 3.11	Python Software Foundation	https://www.python.org/
OpenCV	OpenCV team	https://opencv.org/
pySerial	pySerial	https://github.com/pyserial/pyserial
MATLAB 2021a	The MathWorks, Inc.	https://www.mathworks.com/
Munkres assignment algorithm	Yi Cao	https://www.mathworks.com/matlabcentral/fileexchange/20328-munkres-assignment-algorithm
Other		
Insect collection cage	Bug Dorm	4S1515
Clear acrylic	McMaster	8560K171
White LED light source	Outerbanks Provisions	N/A
A4988 motor driver	StepperOnline	N/A
Arduino Uno R3	Elgoo	N/A
Varifocal USB webcam	ELP	ELP-USBFHD06H-MFV(5-50)
IR filter	Heliopan	703665
IR LED lights	Waveform	7031.85
Compact spectrometer	ThorLabs	CCS100
Light meter	Extech	WBB1642198
Light power meter	ThorLabs	PM100D
Quartz glass capillaries	Sutter Instrument	QF100-70-10
Real-time PCR system	Bio-Rad	CFX96
Micro-injector	Eppendorf	FemtoJet 4i
TetraMin tropical granules	Tetra	16122
Defibrinated sheep blood	Hemostat	DSB1
Zeiss SteREO microscope	Carl Zeiss Microscopy	Discovery.V8
Membrane blood feeder system	Hemotek	SP6W1-3

## Highlights

- *Aedes aegypti* can evade a threatening shadow under very dim light while host seeking
- A rapidly moving shadow is more aversive to mosquitoes than a slowly moving shadow
- Loss of two opsins, Op1 and Op2, diminishes shadow avoidance under dim light only
- Elimination of the TRP channel compromises ability to evade shadows under bright light only
