# Supplementary material for: Visual threat avoidance while host seeking by Aedes aegypti mosquitoes
Source: Cell Rep. Author manuscript; Available in PMC 2025 May 14. (PMC12077400; doi:10.1016/j.celrep.2025.115435)
Supplement: 1 [file NIHMS2076695-supplement-1.pdf]

Cell Reports, Volume *44*

Supplemental information

Visual threat avoidance while host seeking by *Aedes aegypti* mosquitoes

Geoff T. Meyerhof, Pratik Dhavan, Summer Blunk, Allison Bourd, Ramandeep Singh, Avinash Chandel, and Craig Montell

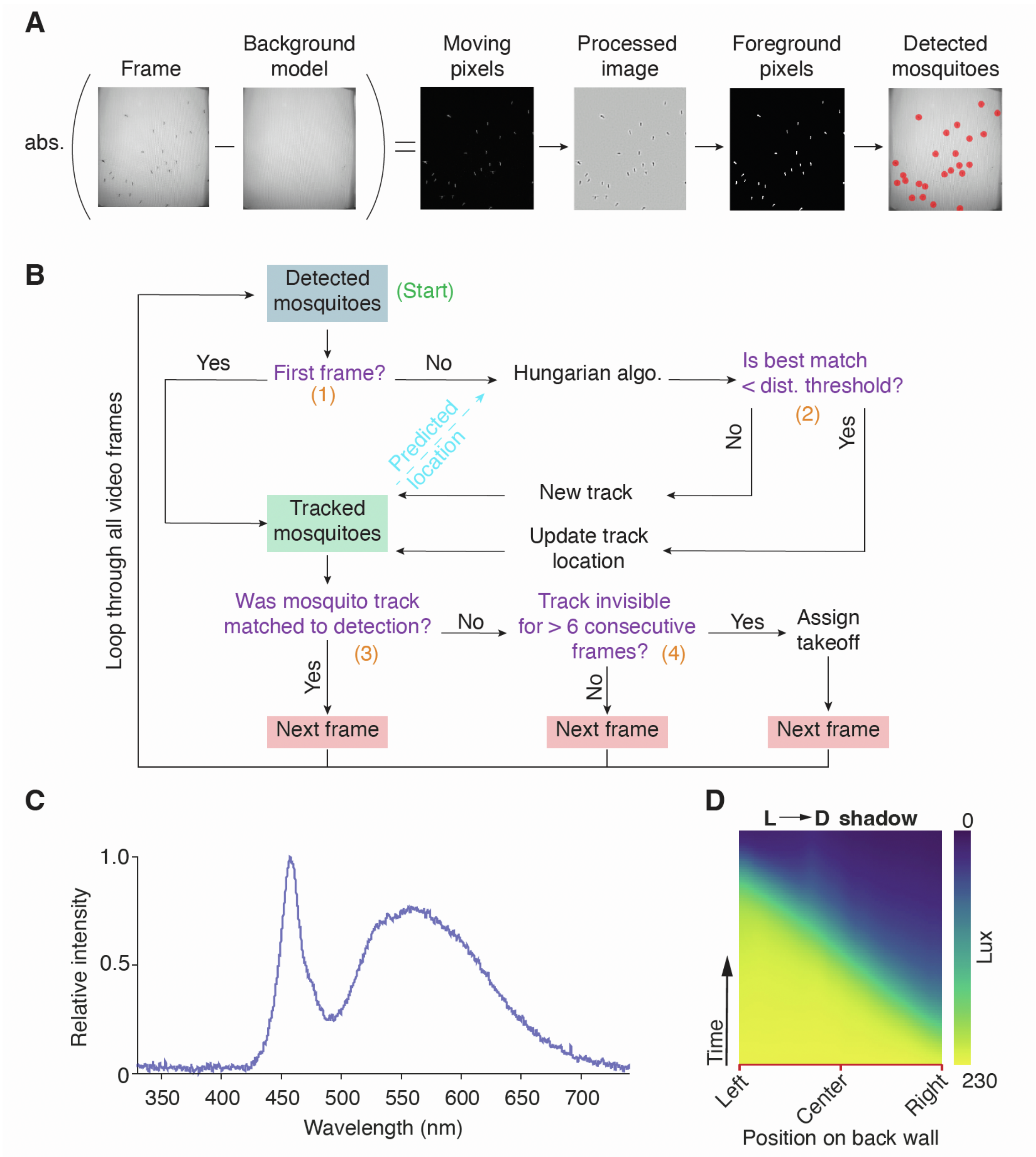

Figure S1. Video tracking methods, light spectrum, and shadow parameters. Related to Figures 1, 2, 4, 5, and 7.

(A) Method for mosquito detection used in automated behavioral tracking. The absolute difference (abs.) between the background model (no mosquitoes present) and frame (mosquitoes present) was determined to identify moving pixels. The moving pixels frame was then processed (Processed image), which served to separate partially overlapping mosquitoes. The processed image was thresholded to identify blobs (i.e.-landed mosquitoes; Foreground pixels). We then stored the centroid coordinates of each mosquito for matching (Detected mosquitoes).

(B) Flow chart of mosquito-matching algorithm for matching mosquitoes between frames. Detected mosquitoes (i.e. blob centroid coordinates) are matched from frame to frame. Tracks without a match for >6 frames are deemed to be takeoff events. See STAR Methods for complete description of (A and B) (Video tracking of mosquitoes).

(C) Spectrum of LED light source used for all behavioral experiments.

(D) Kymograph of a light-to-dark shadow along the back wall of the mosquito cage. During shadow movement events, the mosquitoes experience a rapid change in light intensity following a gradient.

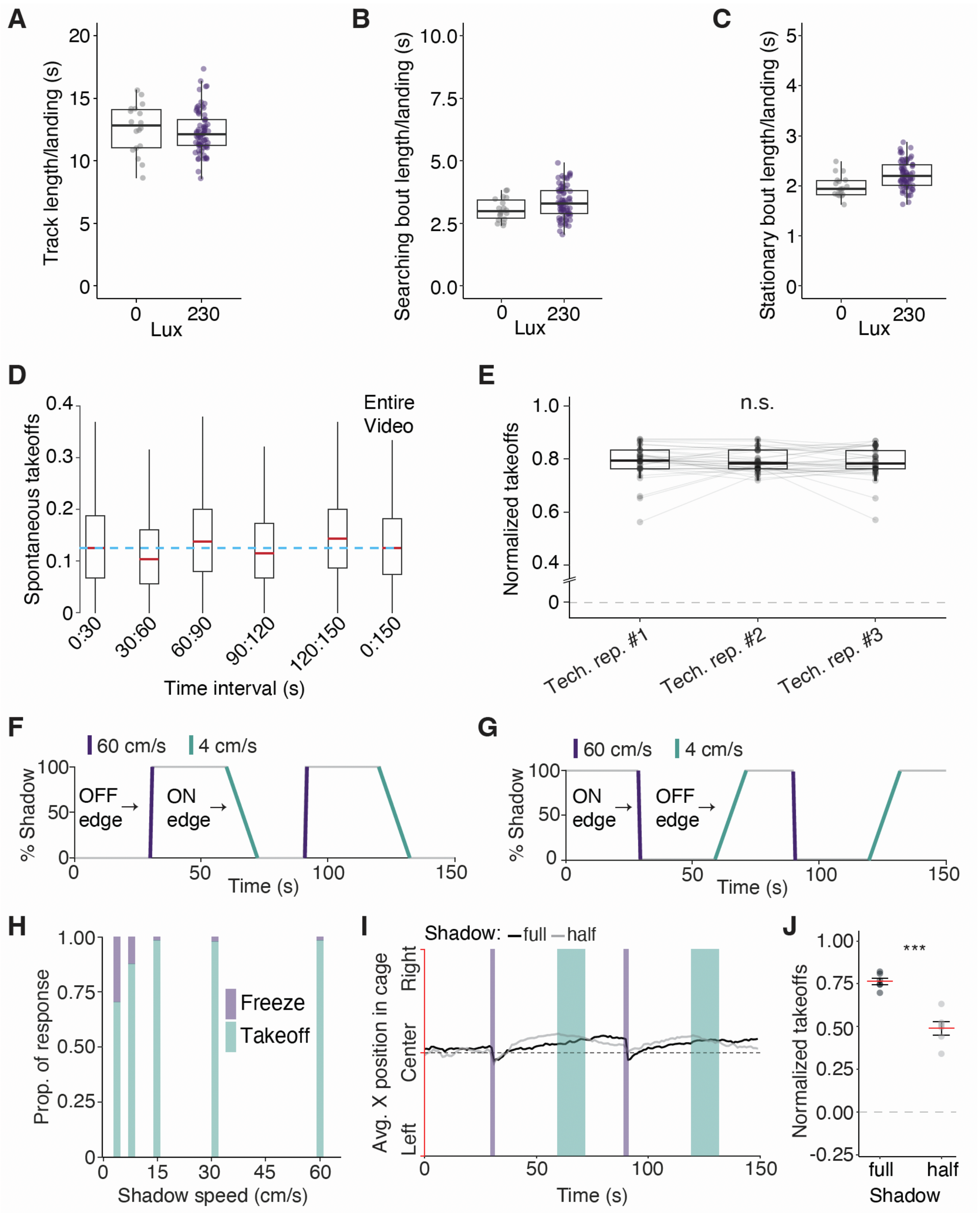

Figure S2. Effect of light on track parameters, spontaneous takeoffs by time, and half-shadow responses. Related to Figures 2 and 4.

(A) Average track length during the behavioral experiments with 0 and 230 lux light.

(B) Average searching bout length per landing during the behavioral experiments under 0 and 230 lux light.

(C) Average stationary bout length per landing during the behavioral experiments under 0 and 230 lux light. n=18 trials from 6 cages with 30 mosquitoes for 0 lux experiments and n=72 trials from 12 cages with 30 mosquitoes for 230 lux experiments.
The boxes in (A–C) indicate the 25th, 50th, and 75th percentiles, with the whiskers extending to maximum/minimum values 1.5 times the interquartile range.
(D) Effect of sampling window on spontaneous takeoff rates. Five, 30-second-long sampling windows were selected to test whether the proportion of mosquitoes showing spontaneous takeoffs during the indicated time windows changed throughout a video. Sampling windows excluded the times during a fast OFF-edge shadow. The mean proportion of mosquitoes taking off during these windows were 0.13, 0.11, 0.15, 0.12, and 0.15. The mean takeoffs spanning the 150 second recording was 0.13. Data for each time window was generated from 50,000 randomly-selected samples from 72 separate recordings.
(E) Normalized takeoffs from technical replicates. Retrials of the same cage were separated by ~22 minutes. n=36 cages of wild-type mosquitoes. One-way ANOVA ($P>0.05$).
The boxes in (D and E) indicate the 25th, 50th, and 75th percentiles, with the whiskers extending to maximum/minimum values 1.5 times the interquartile range.
(F and G) Example shadow movement protocols for testing: (F) OFF-edge shadows, and (G) ON-edge shadows. The velocities of the shadows at 30 and 90 seconds (purple lines) were modified for the variable shadow speed experiments in Figure 4F.
(H) Proportion of response from shadow speed experiments in Figure 4F. Of the mosquitoes that responded to the light-to-dark shadow, the proportion that froze (stopped a searching bout) or initiated a takeoff are indicated.
(I) Average X position of mosquitoes along the back wall of the cage from experiments with full- and half-shadow coverage. Purple vertical lines indicate 60 cm/sec light to dark (OFF-edge) shadows. Green vertical lines indicate 4 cm/sec dark to light (ON-edge) shadows.
(J) Normalized takeoffs from full- and half-shadow experiments (60 cm/sec shadow). Data compared via Wilcoxon Rank-Sum Test. ***$P<0.001$. Means ± SEMs.
(H–J) n=18 trials from 6 separate cages of 30 mosquitoes per condition.

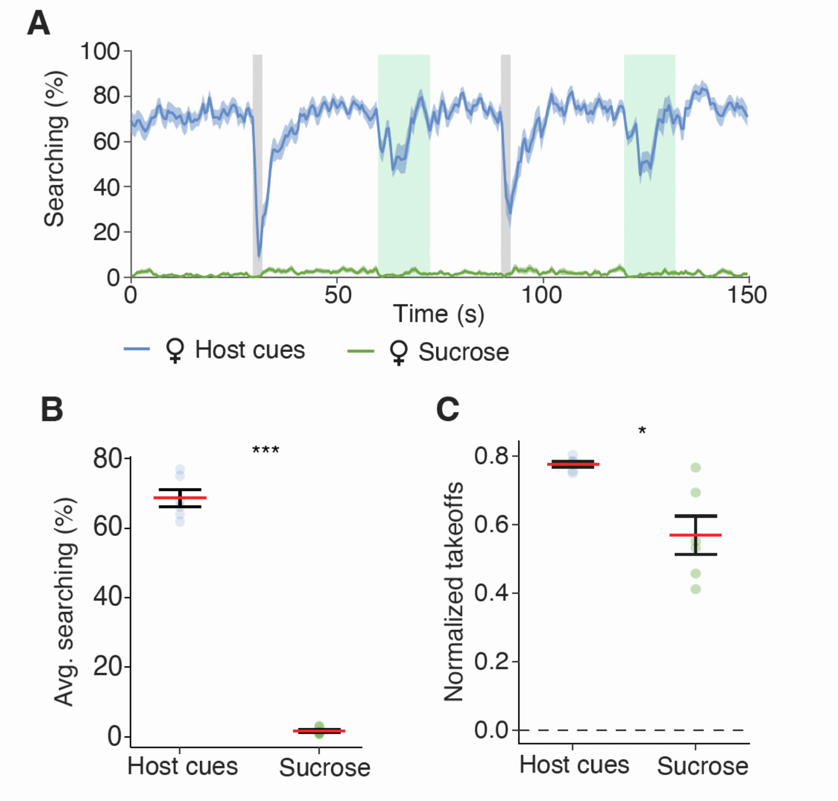

Figure S3. Males and non-host-seeking females takeoff in response to a moving shadow. Related to Figure 5.

(A) Average percentage of landed mosquitoes searching along the back wall. Blue trace, starved females with access to host cues. Green trace, starved females with access to sucrose. Shading over traces indicates SEMs. Grey vertical shading indicates 60 cm/s light-to-dark shadow. Green vertical shading indicates 4 cm/s dark-to-light shadow.

(B) Average percentage of landed mosquitoes searching over the span of the 150-second recording.

(C) Normalized takeoffs in response to 60 cm/s light-to-dark shadow.

n=6 cages of mosquitoes/genotype, each measured three times. Two-tailed unpaired Student's *t*-tests. **P*<0.05. ****P*<0.001. Means ± SEMs.

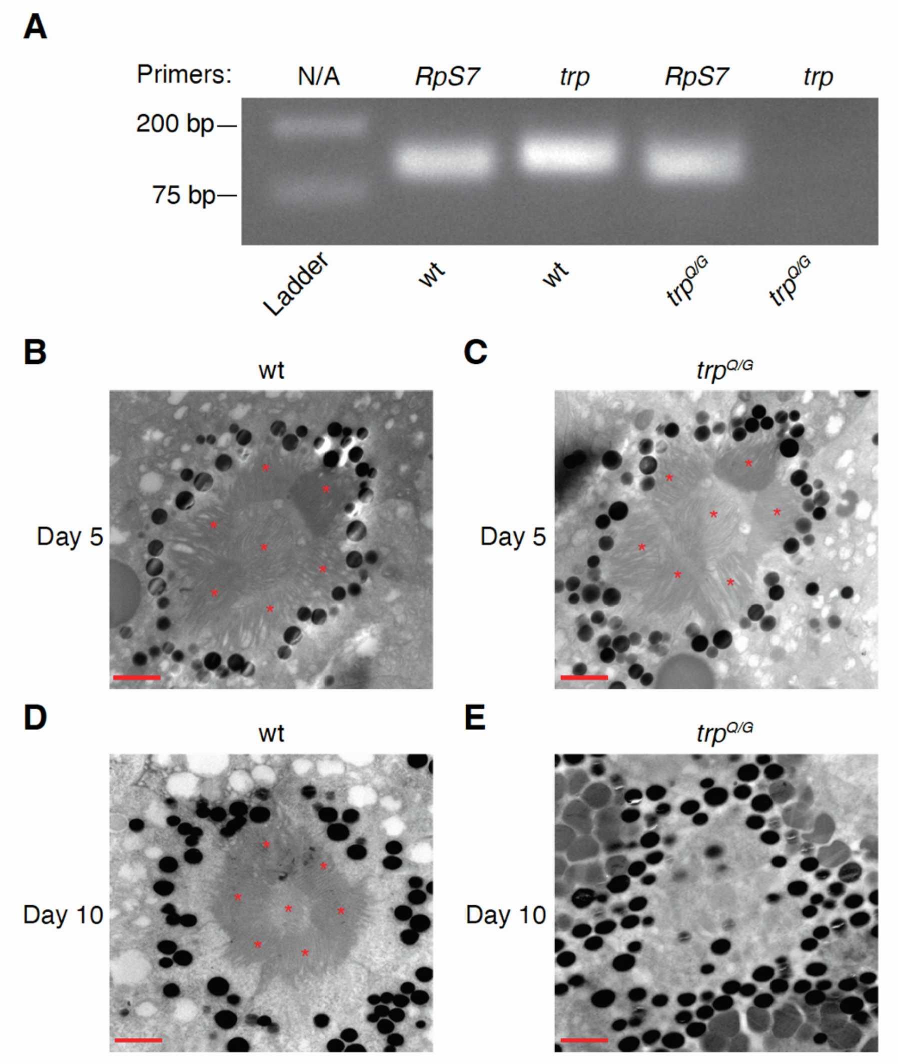

Figure S4. Gel image from RT-qPCR experiment measuring *trp* expression, and TEM images of compound eyes. Related to Figure 6.

(A) Gel image from RT-PCR products measuring *RpS7* mRNA expression and *trp* mRNA expression in wild-type (wt) and $trp^{Q/G}$ mosquitoes.

(B–E) TEM images of sections from the distal regions of retinas obtained from 5- and 10-day-old wt and $trp^{Q/G}$ female mosquitoes. n=3 for all ages and genotypes. (B) 5-day-old wt. (C) 5-day old $trp^{Q/G}$. (D) 10-day-old wt. (E) 10-day-old $trp^{Q/G}$. Scale bars, 2 μm.

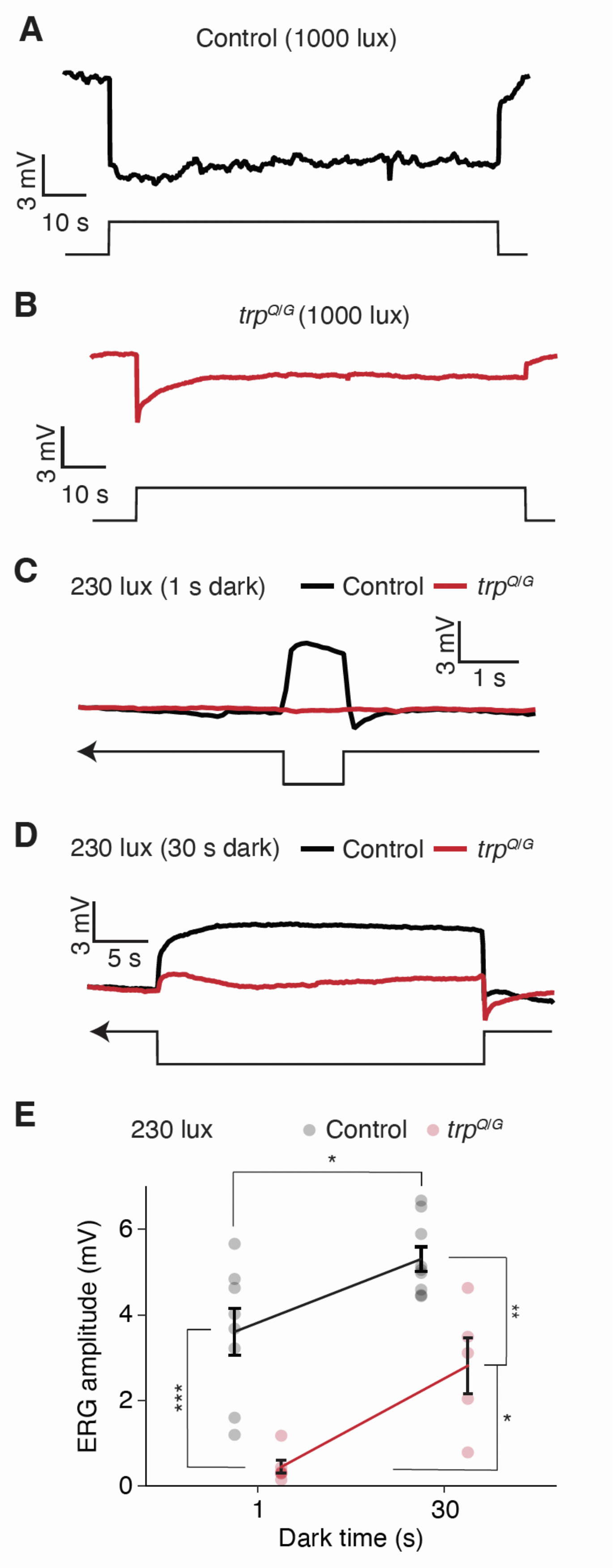

Figure S5. ERG responses to long (90 second) light pulses, and ERG paradigm mimicking behavioral paradigm. Related to Figure 7.

(A and B) Representative ERG traces from one-day-old control mosquitoes exposed to 90 seconds of 1000 lux light. (A) Control. (B) *trp*$^{Q/G}$. n=6.
(C and D) Representative ERG traces from five-day-old mosquitoes exposed to 7 minutes of light followed by one second of dark (C) or 30 seconds of dark (D), and then re-exposed to light. n=5–9.
(E) Quantification of ERG amplitudes from mosquitoes dark adapted for either one or 30 seconds. Two-way ANOVA with factors of dark time, genotype, and genotype:dark time interaction, followed by Tukey's HSD test. n=5–9. **P*<0.05. ***P*<0.01. ****P*<0.001. Means ± SEMs.

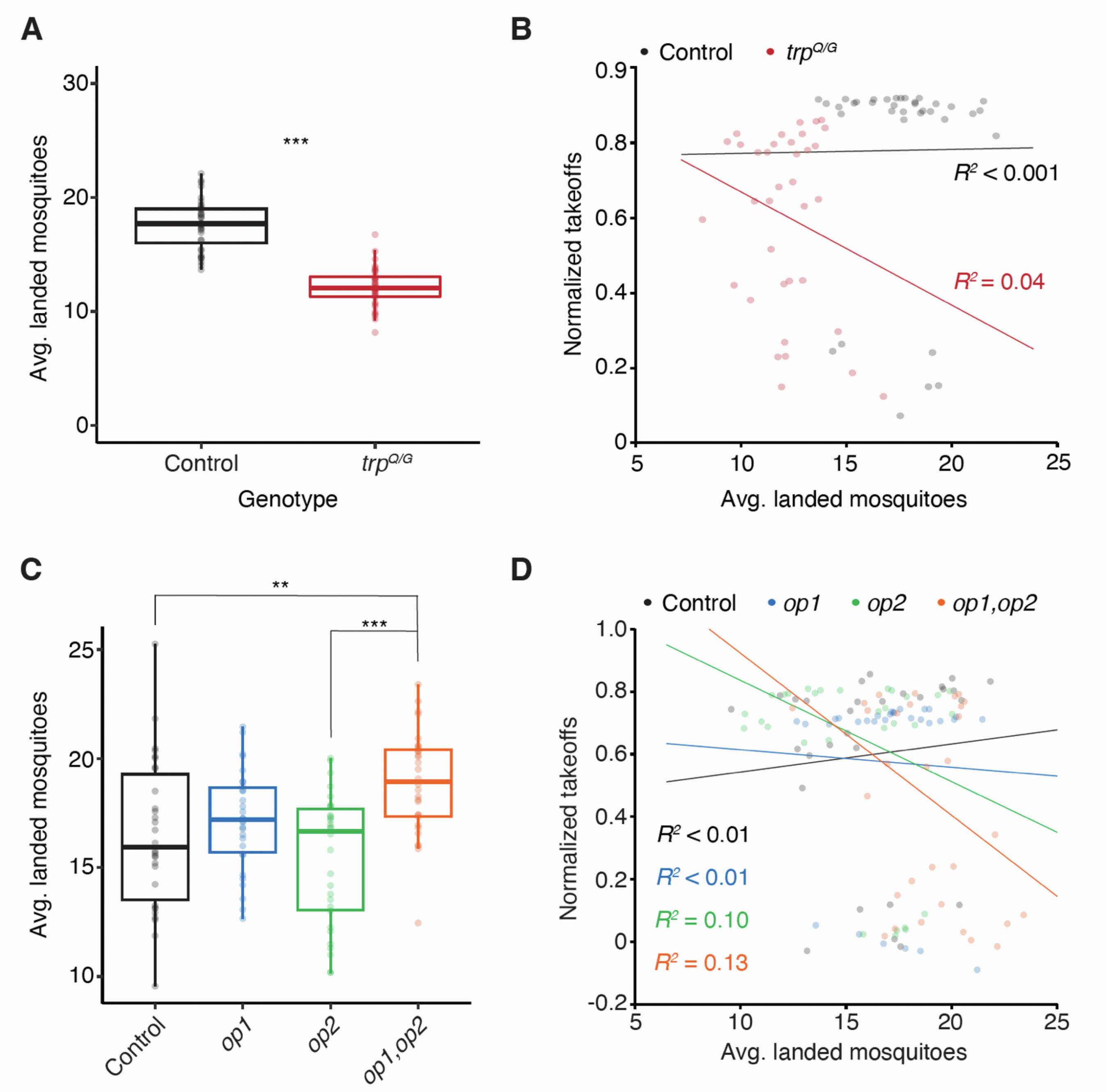

Figure S6. Participation rate is uncorrelated with shadow avoidance. Related to Figure 7.
(A) Participation rate (Avg. landed mosquitoes) of control and *trp*$^{Q/G}$ mosquitoes. Two-tailed Student's *t*-test. The boxes indicate the 25th, 50th, and 75th percentiles, with the whiskers extending to maximum/minimum values 1.5 times the interquartile range.
(B) Correlation between participation rate (Avg. landed mosquitoes) and normalized takeoffs of control and *trp*$^{Q/G}$. Lines indicate linear best fit.
(A–B) Data correspond to Figure 7B. n=36 cages of mosquitoes/genotype.
(C) Participation rate (Avg. landed mosquitoes) of control and opsin mutants. One-way ANOVA, followed by Tukey's HSD test. The boxes indicate the 25th, 50th, and 75th percentiles, with the whiskers extending to maximum/minimum values 1.5 times the interquartile range.
(D) Correlation between participation rate (Avg. landed mosquitoes) and normalized takeoffs of control and opsin mutants. Lines indicate linear best fit.
(C–D) Data correspond to Figure 7E. n=30 cages/genotype. **P<0.01. ***P<0.001.

Oligonucleotides	Source
trp^{QF2} sgRNA sequence:<br>5′-TTCCGTCGCAAGTCACCCGTCGG-3′	This paper
trp^{GFP} sgRNA sequence:<br>5′-ACCAAACACTCCGCTAGGCCTGG-3′	This paper
trp^{QF2} upstream homology arm:<br>F: 5′-CATTTCCTTACCTAGTCCCGACCCAGAGCTTCAGAAC-3′<br>R: 5′-TCCGCTTCCGCCTAGGGGTGACTTGCGACGGAAAC-3′	This paper
trp^{QF2} downstream homology arm:<br>F: 5′-ACGGTATCGATAAGCGTCGGTCAGATCACGCAG-3′<br>R: 5′-ATTTCATTCGCTAGCCCCGAGGGATACCTGTGGATG-3′	This paper
trp^{GFP} upstream homology arm:<br>F: 5′-GATTTCATTCGCTAGCGTGTAGCGACCGTGAAG-3′<br>R: 5′-ATCGATAAGCGCTAGCGCCTGGTAGCGATCCTCGT-3′	This paper
trp^{GFP} downstream homology arm:<br>F: 5′-TCTTAACGCGAGTTAATTAACTAGCGGAGTGTTTGGTCATATTGTACA-3′<br>R: 5′-GCGCACATTTCCTTAATTAAGGGTAACGTATCGCCGTCT-3′	This paper
trp^{QF2} genotyping primers:<br>F: 5′-TAGACAACTGGGAACCGGGG-3′<br>R: 5′-GCGATTTGATCTCGTGCCAT-3′	This paper
trp^{GFP} genotyping primers:<br>F: 5′-TCTTTGCAGTGTTGCTGGGA-3′<br>R: 5′-AACTGATCCCACTGCTCACG-3′	This paper
trp RT-qPCR primers:<br>F: 5′-CGGAAAGAACGAGGATCGCTA-3′<br>R: 5′-TACCAGGACGTAAACCGCAAA-3′	This paper
Rps7 RT-qPCR primers:<br>F: 5′-TACAAGAAGCTGACCGGA-3′<br>R: 5′-TTCCGCGCGCGCTCACTTATTAGATT-3′	This paper
op1^{2} genotyping primers:<br>F: 5’-ACCGCAAGCAACACTTTACG-3’<br>R: 5’-CAAGCGATTTATATTTAGAT-3’	Zhan et al.[S1]
op2^{1} genotyping primers:<br>F:5’-AAGTTATCAGCAAAAGTATC-3’<br>R: 5’-ACCGTGTACGTTCTGTAGC-3’	Zhan et al.[S1]

Table S1: List of oligonucleotide sequences. Related to STAR Methods.

Supplemental Reference

S1 Zhan, Y., Alonso San Alberto, D., Rusch, C., Riffell, J. A. & Montell, C. Elimination of vision-guided target attraction in *Aedes aegypti* using CRISPR. *Curr. Biol.* 31, 4180-4187 (2021).
